# Brain region–specific lipid alterations in the PLB4 hBACE1 knock-in mouse model of Alzheimer’s disease

**DOI:** 10.1186/s12944-020-01367-8

**Published:** 2020-08-31

**Authors:** Madhurima Dey, Frank J. Gunn-Moore, Bettina Platt, Terry K. Smith

**Affiliations:** 1 School of Biology, University of St. Andrews, Medical & Biological Sciences Building, St. Andrews, Fife, Scotland; 2 School of Medicine, Medical Sciences & Nutrition, University of Aberdeen, Institute of Medical Sciences, Aberdeen, Scotland; 3 Biomedical Science Research Complex, University of St. Andrews, St. Andrews, Fife, Scotland

**Keywords:** PLB4, Quantitative lipidomics, Brain regions

## Abstract

**Background:**

Lipid dysregulation is associated with several key characteristics of Alzheimer’s disease (AD), including amyloid-β and tau neuropathology, neurodegeneration, glucose hypometabolism, as well as synaptic and mitochondrial dysfunction. The β-site amyloid precursor protein cleavage enzyme 1 (BACE1) is associated with increased amyloidogenesis, and has been affiliated with diabetes via its role in metabolic regulation.

**Methods:**

The research presented herein investigates the role of hBACE1 in lipid metabolism and whether specific brain regions show increased vulnerability to lipid dysregulation. By utilising advanced mass spectrometry techniques, a comprehensive, quantitative lipidomics analysis was performed to investigate the phospholipid, sterol, and fatty acid profiles of the brain from the well-known PLB4 hBACE1 knock-in mouse model of AD, which also shows a diabetic phenotype, to provide insight into regional alterations in lipid metabolism.

**Results:**

Results show extensive region – specific lipid alterations in the PLB4 brain compared to the wild-type, with decreases in the phosphatidylethanolamine content of the cortex and triacylglycerol content of the hippocampus and hypothalamus, but increases in the phosphatidylcholine, phosphatidylinositol, and diacylglycerol content of the hippocampus. Several sterol and fatty acids were also specifically decreased in the PLB4 hippocampus.

**Conclusion:**

Collectively, the lipid alterations observed in the PLB4 hBACE1 knock-in AD mouse model highlights the regional vulnerability of the brain, in particular the hippocampus and hypothalamus, to lipid dysregulation, hence supports the premise that metabolic abnormalities have a central role in both AD and diabetes.

## Background

The brain contains the second highest lipid content of all the organs in the human body [1], where the compositional diversity of lipids and the wide ranging lipid-lipid and lipid-protein interactions have been associated with various functions ranging from axonal regeneration [2] and synaptogenesis [3] to synaptic plasticity [4]. At a structural level, phospholipids (with their varied fatty acid tails) and sterols within the bilayer of cell membranes form lipid rafts, which are specialised, highly structured regions that act as a microdomain for the regulation of specific transmembrane proteins, such as the amyloid precursor protein (APP), and give rise to lateral inhomogeneity within the phospholipid bilayer [5].

Spatial and temporal lipid homeostasis is a tightly regulated process, and increasing evidence for aberrant lipid metabolism due to the dysregulation of homeostatic mechanisms has been linked to metabolic diseases, such as diabetes [6], the lysomal storage disorder Niemann–Pick disease type C [7], as well as neurological disorders including major depression, bipolar disorder [8], schizophrenia [9], and a range of neurodegenerative disorders including Parkinson’s disease [10], dementia with Lewy bodies [11], and Alzheimer’s disease (AD); the focus of the research presented herein.

Lipids, and their associated fatty acids, play a key role in energy metabolism under glucose hypometabolism circumstances, as associated within the AD brain, where the compensatory shift to alternative energy substrates, such as ketone bodies, are thought to be derived from the beta-oxidation catabolism of fatty acids from neutral lipids and structural lipids [12].

The role of lipid abnormalities in the pathogenesis of AD is emphasised via the association of genes involved in lipid transport, processing, and metabolism, such as the ε4 allele of apolipoprotein E (APOE), which is the strongest genetic risk factor for both sporadic early- and late-onset AD [13]. The APOE gene encodes the major cholesterol transporter protein that shuttles cholesterol, as well as other lipids, between cells in an isoform-dependent manner [14]. The APOE protein is expressed in the periphery and the central nervous system (CNS), predominantly in astrocytes and microglia, but also in neurons under stress conditions [15], but interestingly APOE does not appear to cross the blood-brain-barrier (BBB), so the peripheral and CNS pools of the protein are considered to be independent of each other [16]. However recent studies suggest that different *APOE* statuses can promote disease through different mechanisms, dependent upon a leaky blood-brain barrier [17].

The importance of understanding and delineating the mechanisms contributing to aberrant lipid homeostasis in AD is highlighted by research suggesting the use of lipid biomarkers to distinguish between mild cognitive impairment (MCI) and AD [18–20], but further research is needed to gain a more detailed understanding of the biochemical and mechanistic alterations associated with the lipid dysregulation observed in AD.

The recent PLB4 transgenic AD mouse model, based upon the targeted neuronal knock-in of human BACE1 (hBACE1) on an endogenous mouse BACE1 background, recapitulates an ‘AD-like’ phenotype with age-dependent Aβ accumulation, cognitive deficits and cerebral hypometabolism, as well as a diabetic phenotype [21]. The role of BACE1 as the rate-limiting step of Aβ_40/42_ production [22], together with research showing increased protein levels and enzymatic activity of BACE1 in AD [23] make it an appealing therapeutic target [24]. However, BACE1 has many physiological roles, for example in metabolism [25], the regulation of astrogenesis and neurogenesis via Notch signalling [26], myelination [27], and synaptic function [28], which complicates the potential for inhibiting the BACE1 enzyme, and emphasises the need for further research into the role of BACE1. Previous research indicates that APP may be present in two cellular pools – one associated with lipid rafts, which preferentially favours the amyloidogenic cleavage by BACE1 (β-secretase) and γ-secretase, producing Aβ_40/42_, the other in non-raft regions, where it is cleaved by α-secretase in the non-amyloidogenic pathway [29]. Therefore, lipid dysregulation and alterations in the cell membrane composition coupled with increased BACE1 activity could contribute to the increased deposition of Aβ_40/42_ within the brain. Insight into the specific lipid groups influenced by BACE1 would thus provide a mechanistic understanding of lipid alterations that may be linked to AD-relevant phenotypes. Studies by Plucińska et al. previously explored the global phospholipid profile of the PLB4 forebrain and plasma in the context of diabetes to show that PE and PS were predominantly increased in the forebrain and plasma samples of the PLB4 mouse model compared to the wild type (PLB_WT_) [25].

This study applies a range of combinatory mass spectrometry techniques to characterise the key alterations in lipid profiles associated with the neuronal hBACE1 knock-in. The phospholipid, sterol, and fatty acid content of the cortex, hippocampus, and hypothalamus of the PLB4 mouse brain was analysed to gain insight into the downstream effects of the hBACE1 expression, and furthermore to investigate whether the PLB4 mouse brain shows regional vulnerability to lipid dysregulation.

## Methods

The aim of this study was to investigates the role of hBACE1 in lipid metabolism and whether specific brain regions show increased vulnerability to lipid dysregulation. By utilising advanced mass spectrometry techniques, this study presents a comprehensive, quantitative lipidomics analysis of the phospholipid, sterol, and fatty acid profiles of the brain from the well-known PLB4 hBACE1 knock-in mouse model of AD, which also shows a diabetic phenotype, to provide insight into regional alterations in lipid metabolism.

### PLB4 hBACE1 knock-in mouse model

The PLB4 hBACE1 mouse model was generated by the targeted knock-in of a single copy of the human *bace1* (*hbace1*) cDNA construct, on an unaltered endogenous *bace1* ^+/+^ background in C57BL/6 J blastocytes, as detailed in Plucińska et al. [21]. The gene expression was controlled by the mouse CaMKIIα promoter, cloned into the HPRT locus, to ensure a neuron- and forebrain-specific expression of the transgene. PLB_WT_ control mice were generated via the same knock-in technique [30]. All animals were housed and tested in accordance with European (European Directive on the protection of animals used for scientific purposes; 2010/63/EU) and UK Home Office regulations, experiments were approved by the University Ethics Board and performed in accordance with the Animal (Scientific Procedures) Act 1986. Mice were bred at Charles River UK and shipped to facilities at the University of Aberdeen at least one week prior to tissue harvest. Animals were housed with ad libitum access to water and food, with a circadian regime of 12 h.

The hBACE1 expression profile in PLB4 mice was confirmed by Southern blot, western blot, and immunohistochemical analysis [21], with no difference in expression detected between males and females. An AD-relevant phenotype was the most pronounced at 6 months of age in the PLB4 mice, displaying several memory impairments, as well as both intra- and extracellular accumulation of amyloid-β throughout the forebrain, accompanied by the corresponding reduction of full-length APP in the PLB4 brain (− 29% relative to PLB_WT_) [21, 30].

### Lipid extraction

The cortex, hippocampus, and hypothalamus were sub-dissected on ice from PLB_WT_ controls and PLB4 mouse brains (*n* = 6; male; age: 6 months) and snap frozen in liquid nitrogen. Lipid extractions (all solvents used were of HPLC grade from Sigma/Aldrich, Dorset, UK) were performed on weighed amounts of tissue at room temperature, according to the methodology of Bligh and Dyer [31]; the tissue was homogenised in a 1 mL glass Dounce homogeniser in methanol, transferred to a glass vial, and chloroform was added to give a 1:2 (*v/v*) ratio of chloroform:methanol. At the point of lipid extraction, ~ 10 μL (normalised to the amount of tissue) of the internal standard, SPLASH® LIPIDOMIX® Mass Spec Standard (suspended 1000 μL) (Avanti Polar Lipids: 330707, AL, USA), was added to each sample for normalisation, and the samples were then mixed on a shaker (Hawksley London: 1287) for 15 min at room temperature. Chloroform and ddH_2_O were added to give a final chloroform:methanol:ddH_2_O ratio of 2:2:1 (*v/v*), and the samples were centrifuged at 1000 g for 5 min at room temperature. The lower phase was transferred to a new glass vial and dried under nitrogen stored at 4 °C until subsequent lipid analysis.

### Sterol analysis

Dried lipid extracts from above were reconstituted in dichloromethane (~ 20 μL) and analysed by gas chromatography - mass spectrometry (GC-MS) for identification of sterols using a 6890 GC 5973 N MSD system and 7683 Series Injector, Agilent Technologies, with a ZB-50 column (15 M × 32 mm ID × 0.5 mm thickness, Phenomenex, Cheshire, UK), with 1 μL splitless injection, and a GC temperature programme of 100 °C for 1 min, increase in gradient up to 200 °C at 8 °C/ min, 200 °C for 2 min, increase in gradient up to 300 °C at 3 °C/ min, and 300 °C for 15 min. Sterols were identified by comparison of retention time and fragmentation pattern, and normalised to D7-cholesterol as the internal standard, derived from the previously added SPLASH® LIPIDOMIX® Mass Spec Standard (Avanti Polar Lipids: 330707).

### Total fatty acid analysis

Fatty acids within the lipid extracts were transmethylated to fatty acid methyl esters (FAMEs) by esterification for analysis by GC-MS. The dried lipid extract was transmethylated with an 8% solution of HCl in methanol/water (85:15, *v/v*) and toluene, and incubated overnight at 45 °C, following which the methanol was evaporated, samples dried using a Savant™ SPD121P SpeedVac™ Concentrator (Thermo Fisher Scientific), and hexane was added, making the solution biphasic. The hexane layer was extracted and transferred to a new glass vial and dried under nitrogen. The dried sample was reconstituted in dichloromethane and analysed by GC-MS using a 6890 GC 5973 N MSD system and 7683 Series Injector, Agilent Technologies, with a ZB-5 column (30 M × 25 mm × 25 mm, Phenomenex), with 1 μL splitless injection, and a GC temperature programme of 70 °C for 10 min, increase in temperature gradient up to 220 °C at 5 °C/min, and 220 °C for 15 min. FAMEs were identified by comparison of retention time and fragmentation pattern, and normalised to the C15:0 as the internal standard, derived from the previously added SPLASH® LIPIDOMIX® Mass Spec Standard (Avanti Polar Lipids: 330707).

### Phospholipid analysis

Phospholipids were analysed by electrospray ionisation - tandem mass spectrometry (ESI-MS/MS) using the Absceix 4000 QTrap, a triple quadrupole mass spectrometer with a nanoelectrospray source. The dried lipid extracts were reconstituted in 1:2 (*v/v*) chloroform:methanol and 6:7:2 (*v/v*) acetonitrile:isopropanol:water, delivered via a nanomate, and analysed in positive and negative ion modes using a capillary voltage of 1.25 kV. Tandem mass spectra scanning (daughter, precursor, and neutral loss scans) were performed using nitrogen as the collision gas, with collision energies between 35 and 90 V. Assignment of phospholipid species was based upon a combination of survey, daughter, precursor and neutral loss scans; the phospholipid peaks identified from the tandem MS/MS methodologies were identified and verified using the LIPID MAPS Lipidomics Gateway (http://www.lipidmaps.org). Each phospholipid class was normalised to the corresponding internal standard from the SPLASH® LIPIDOMIX® Mass Spec Standard (Avanti Polar Lipids).

### Statistical analysis

Following normalisation of ESI-MS/MS and GC-MS data to the corresponding internal standard, statistical analysis was performed on GraphPad Prism (version 7.00 software, La Jolla California USA) using an unpaired, two-tailed Student’s T-test with Welch’s correction or Wilcoxon signed-rank test to determine statistically significant differences between PLB_WT_ and PLB4 lipid species and brain regions.

## Results

### Sterol alterations in the PLB4 brain

**Fig. 1:**
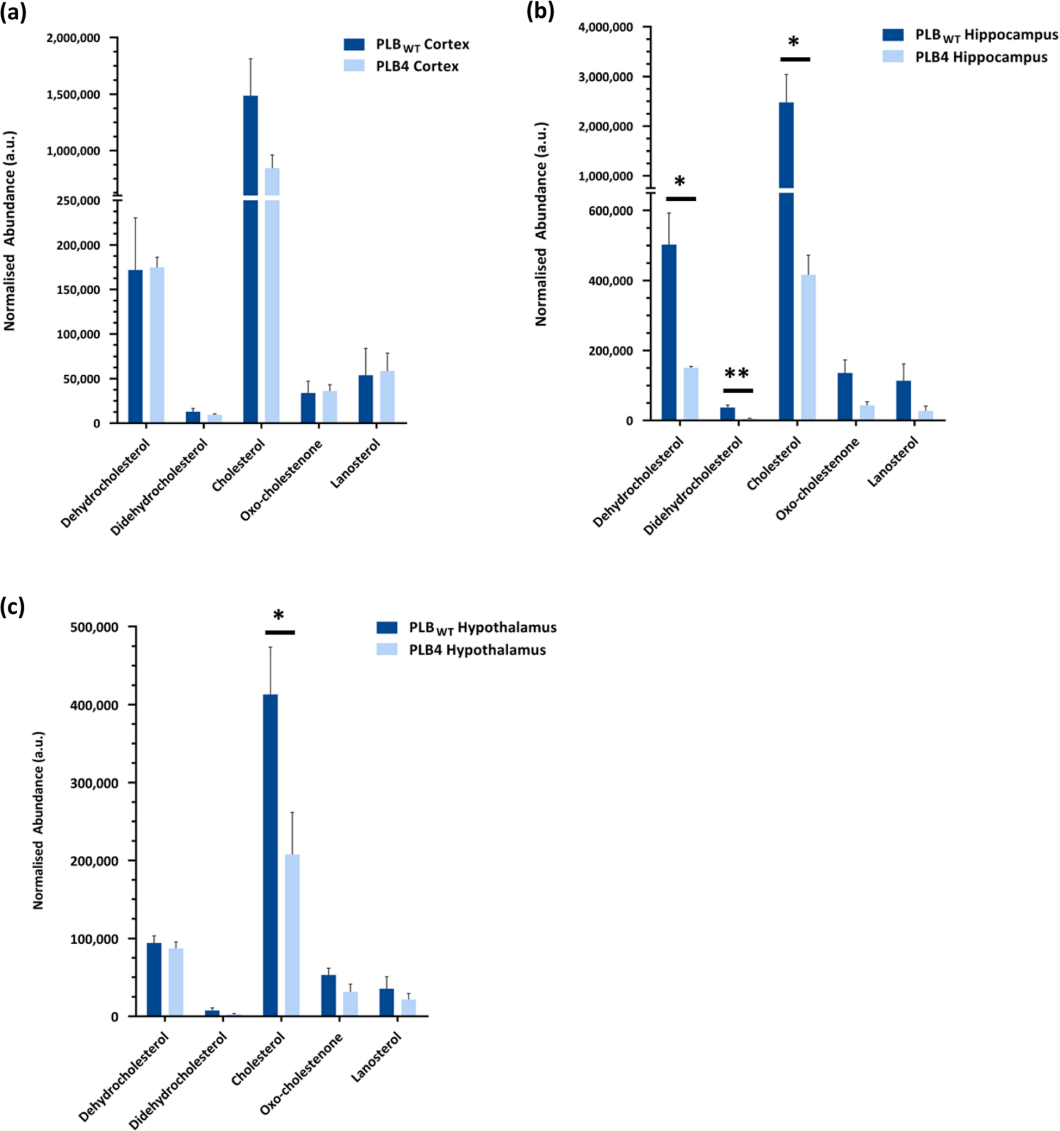
Sterol analysis of the PLB_WT_ and PLB4 brain. The relative abundance of each sterol identified in the cortex (**A**), hippocampus (**B**), and hypothalamus (**C**) was normalised to D7-cholesterol as the internal standard. Annotation of the major sterol species were identified from MS daughter ion fragmentation scans. Data represented as average ± S.E.M (*n* = 6). The normalised abundance (arbitrary units) refers to the normalised total ion count for individual molecular species. An unpaired, two-tailed Student’s t-test with Welch’s correction was performed to determine statistically significant differences between PLB_WT_ and PLB4 data. * *P* ≤ 0.05, ** *P* ≤ 0.01. Cortex: all *p* ≥ 0.05; hippocampus: dehydrocholesterol *P* = 0.0113, didehydrocholesterol *P* = 0.0099, cholesterol *P* = 0.0151; hypothalamus: cholesterol *P* = 0.0326

The analysis of the PLB_WT_ and PLB4 brain regions by gas chromatography - mass spectrometry (GC-MS) enabled the detection and identification of several upstream components within the cholesterol biosynthesis pathway. The abundance of the respective sterols within the cortex, hippocampus, and hypothalamus was determined using GC-MS, which, coupled with the MS fragmentation scan allowed identification of all of the sterol species detected. Normalisation of the respective individual sterol total ion count data to that of D_7_ cholesterol within the internal standard (added at the point of lipid extraction) allowed a quantitative comparison between the PLB_WT_ and PLB4 samples (Fig. 1). The general trend observed throughout the sterol analysis was a decrease in all of the sterol species in the PLB4 brain regions compared to the PLB_WT_ controls. While the cortex (Fig. 1A) did not show any significant decrease in any particular sterol species, statistical decreases in the cholesterol levels were observed in the PLB4 hippocampus and hypothalamus brain regions. The majority of the sterol alterations were in the PLB4 hippocampus (Fig. 1B), with statistically significant decreases seen in dehydrocholesterol (by 3.3-fold, *P* = 0.0133), didehydrocholesterol (by 7.8-fold, *P* = 0.0099), and cholesterol (by 5.9-fold *P* = 0.0151) compared to the PLB_WT_ hippocampus. The most prominent change in the hypothalamus samples was a statistically significant decrease by 2.0-fold (*P* = 0.0326) in the cholesterol content of the PLB4 hypothalamus compared to the PLB_WT_ hypothalamus (Fig. 1C).

### Fatty acid alterations in the PLB4 brain

**Fig. 2:**
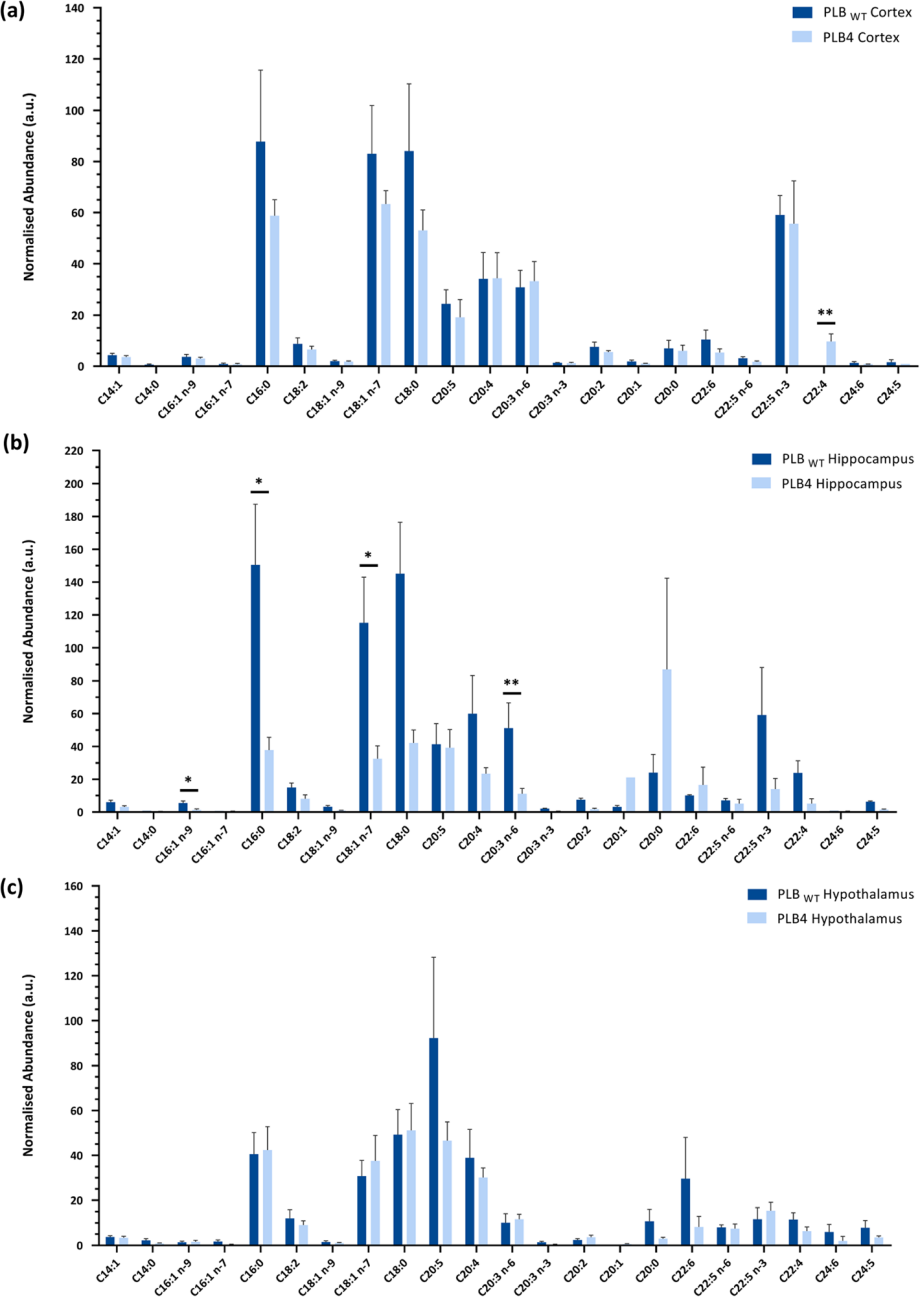
Analysis of the fatty acid species in the PLB_WT_ and PLB4 brain. The relative abundance of each fatty acid methyl ester (FAME) identified in the cortex (**A**), hippocampus (**B**), and hypothalamus (**C**) was normalised to C15:0 as the internal standard. Annotation of the major FAME species were identified from MS daughter ion fragmentation scans**.** Data represented as average ± S.E.M (n = 6). The normalised abundance (arbitrary units) refers to the normalised total ion count for individual molecular species. An unpaired, two-tailed Student’s t-test with Welch’s correction was performed to determine statistically significant differences between PLB_WT_ and PLB4 data. * *P* ≤ 0.05, ** *P* ≤ 0.01; hippocampus FAMEs - C16:1 *P* = 0.0325, C16:0 *P* = 0.0112, C18:0 *P* = 0.0204, C20:3 n-6 *P* = 0.0060

Fatty acids within the lipid extracts were transmethylated to fatty acid methyl esters (FAMEs), which were then analysed by GC-MS. The detection and identification of saturated and unsaturated fatty acids was performed by utilising a combination of the GC retention time and the MS fragmentation pattern comparing with standards. Normalisation of the respective FAME total ion count data to that of C15:0 within the internal standard (added at the point of lipid extraction) allowed a quantitative comparison between the PLB_WT_ and PLB4 samples derived from the different brain regions (Fig. 2A-C). Although a large degree of biological variability was observed between the replicate samples, the highest number of alterations were observed within the hippocampus (Fig. 2B), which showed statistically significant decreases in C16:1 (palmitoleic acid) by 4.2-fold (*P* = 0.0325), C16:0 (palmitic acid) by 4.0-fold (*P* = 0.0112), C18:0 (stearic acid) by 3.5-fold (*P* = 0.0204), and C20:3 n-6 (dihomo-γ-linoleic acid) by 4.6-fold (*P* = 0.0060) in the PLB4 hippocampus compared to the PLB_WT_ hippocampus. The ratio of saturated to unsaturated FAMEs within each brain region was also calculated from the normalised GC-MS data to investigate whether the alterations observed in the PLB4 brain preferentially influenced either type of FAME (Supplementary Fig. S1), but no statistically significant alterations were observed.

### Phospholipid alterations in the PLB4 brain

**Fig. 3:**
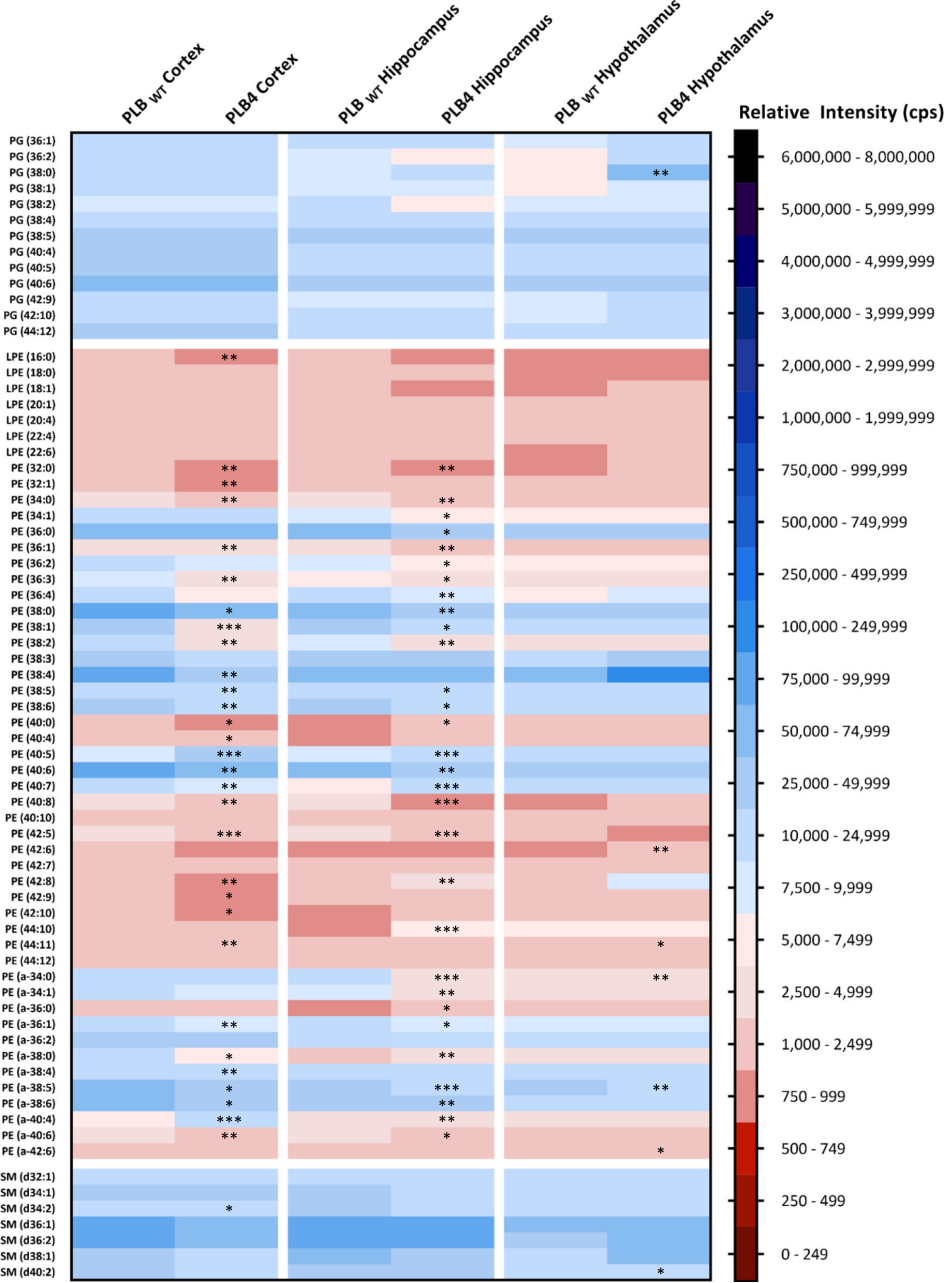
Phospholipid analysis of the PG, PE, and SM content of the PLB_WT_ and PLB4 brain. Analysis of the phosphatidylglycerols (PG), phosphatidylethanolamines (PE), and sphingomyelins (SM) in the PLB_WT_ and PLB4 cortex, hippocampus, and hypothalamus. Each phospholipid class was normalised to its corresponding internal standard. The relative intensity (cps) refers to the normalised total ion count (TIC) for the individual molecular species. Data represented as an average of *n* = 6. An unpaired, two-tailed Student’s t-test with Welch’s correction was performed to determine statistically significant differences between PLB_WT_ and PLB4 data; * *P* ≤ 0.05, ** *P* ≤ 0.01, *** *P* ≤ 0.001

**Fig. 4:**
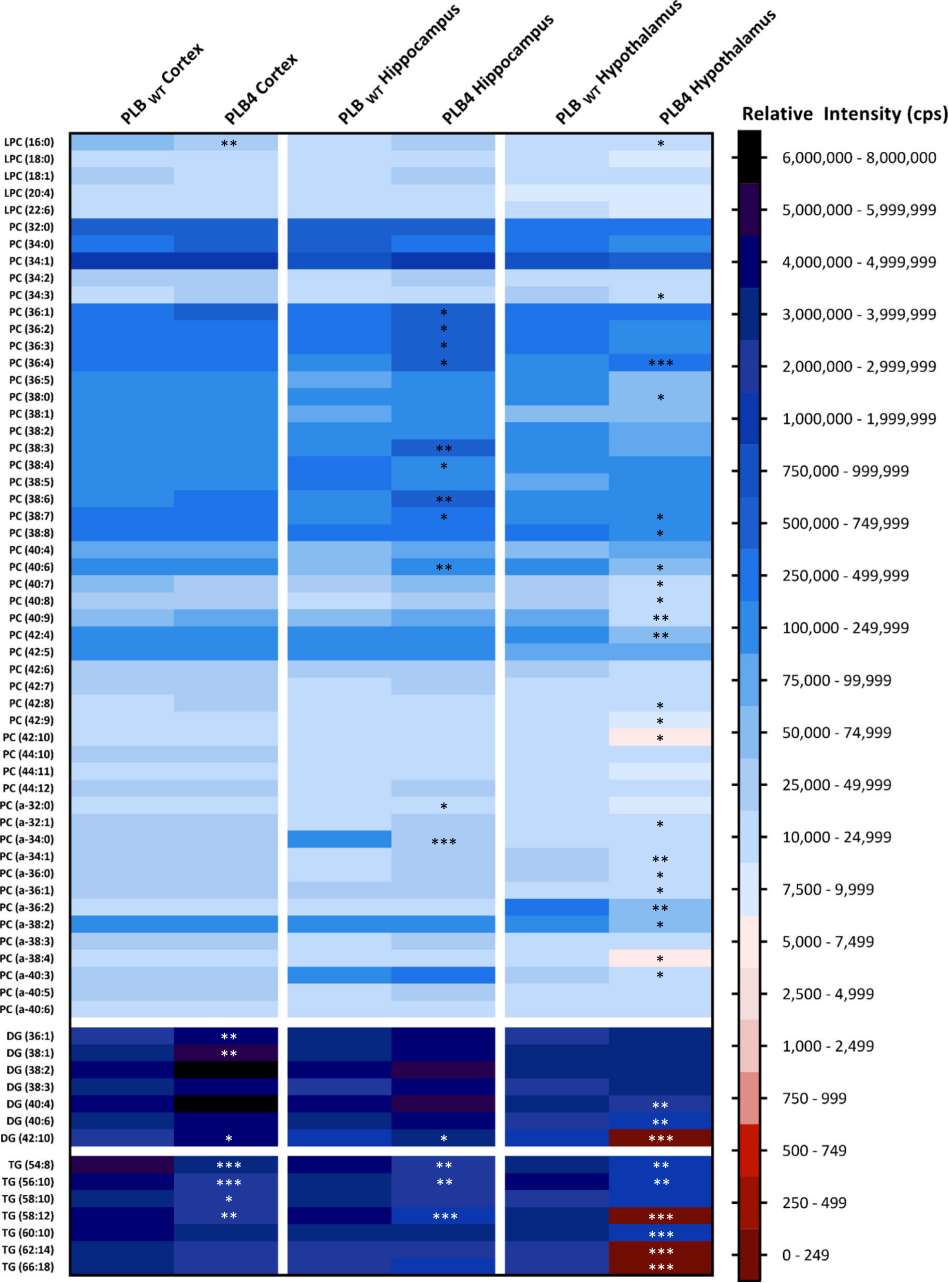
Phospholipid analysis of the PC, DG, and TG content of the PLB_WT_ and PLB4 brain. Analysis of the phosphatidylcholines (PC), diacylglycerols (DG), and triacylglycerols (TG) in the PLB_WT_ and PLB4 cortex, hippocampus, and hypothalamus. Each phospholipid class was normalised to its corresponding internal standard. The relative intensity (cps) refers to the normalised total ion count (TIC) for the individual molecular species. Data represented as an average of n = 6. An unpaired, two-tailed Student’s t-test with Welch’s correction was performed to determine statistically significant differences between PLB_WT_ and PLB4 data; * *P* ≤ 0.05, ** *P* ≤ 0.01, *** *P* ≤ 0.001 (white asterisks used only for visibility purposes)

**Fig. 5:**
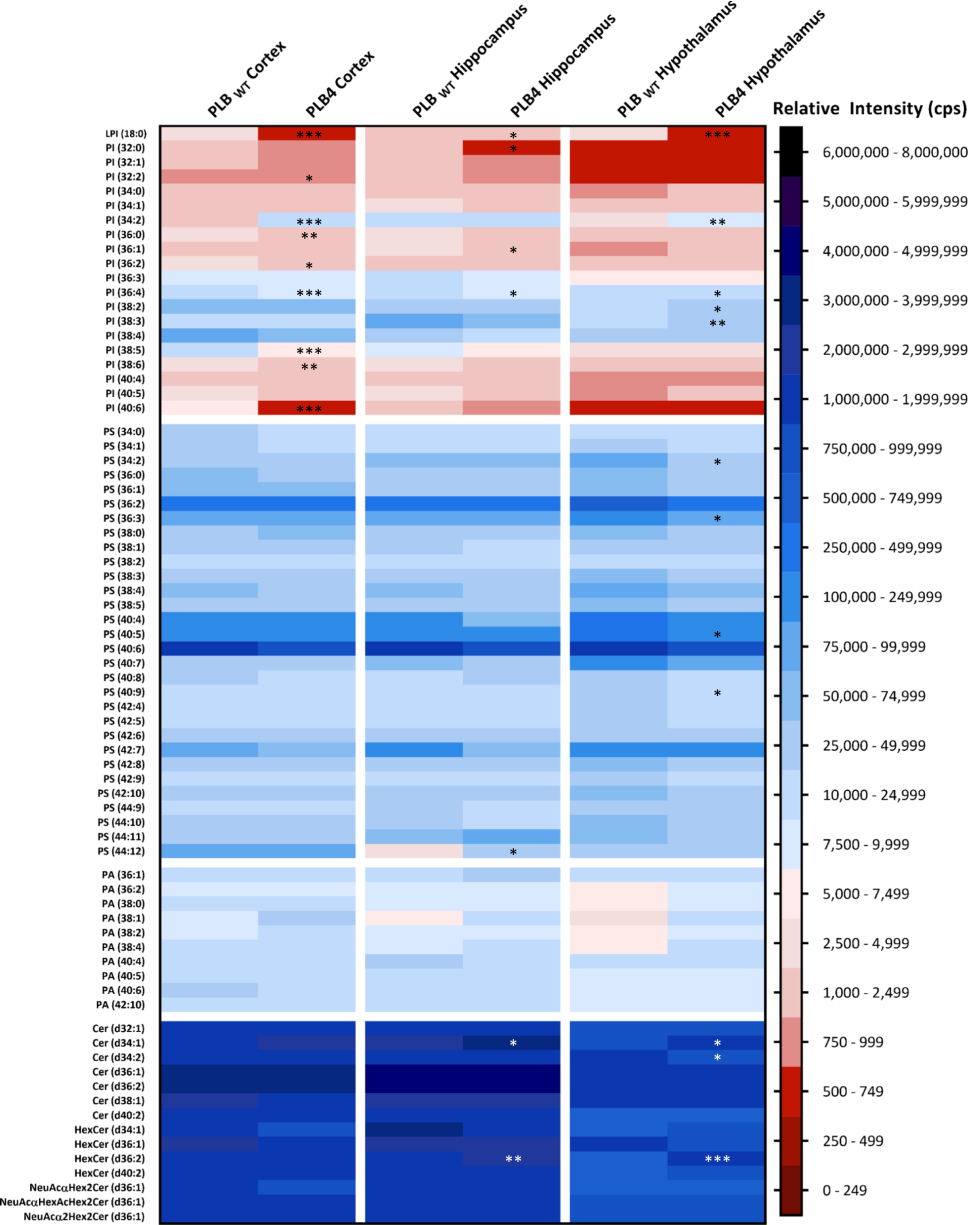
Phospholipid analysis of the PI, PS, PA and Cer content of the PLB_WT_ and PLB4 brain. Analysis of phosphatidylinositols (PI), phosphatidylserines (PS), phosphatidic acids (PA) and ceramides (Cer) in the PLB_WT_ and PLB4 cortex, hippocampus, and hypothalamus. Each phospholipid class was normalised to its corresponding internal standard. The relative intensity (cps) refers to the normalised total ion count (TIC) for the individual molecular species. Data represented as an average of n = 6. An unpaired, two-tailed Student’s t-test with Welch’s correction was performed to determine statistically significant differences between PLB_WT_ and PLB4 data; * *P* ≤ 0.05, ** *P* ≤ 0.01, *** *P* ≤ 0.001 (white asterisks used only for visibility purposes)

**Fig. 6:**
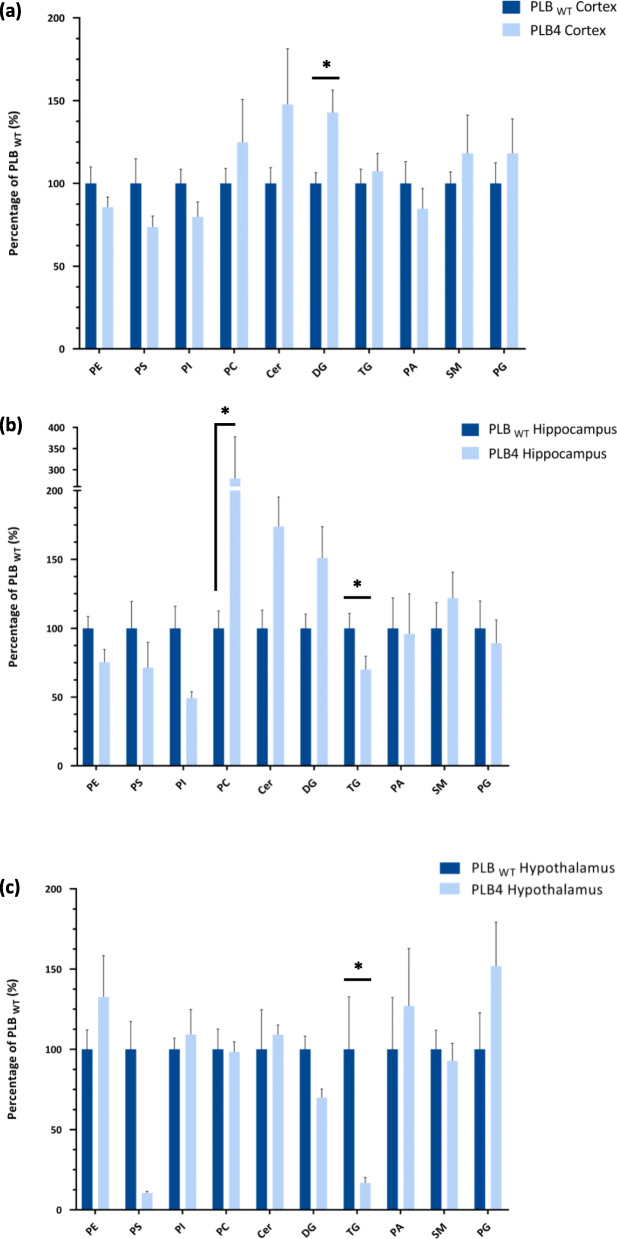
Phospholipid profiles of the PLB_WT_ and PLB4 cortex, hippocampus, and hypothalamus. Each lipid class (sum of all species) is represented as an average percentage (± SEM) of the corresponding PLB_WT_ class, n = 6. A Wilcoxon signed-rank test was performed to determine statistically significant differences between PLB_WT_ and PLB4 data, where * denotes *P* ≤ 0.05. Cortex: PE PLB_WT_ vs. PLB4: *P* = 0.0313; DG PLB_WT_ vs. PLB4: *P* = 0.0313; TG PLB_WT_ vs. PLB4: *p* = 0.0313. Hippocampus: PC PLB_WT_ vs. PLB4: *P* = 0.0313; TG PLB_WT_ vs. PLB4: *P* = 0.0313. Hypothalamus: TG PLB_WT_ vs. PLB4: *P* = 0.0313. Abbreviations: PE – phosphatidylethanolamine, PS – phosphatidylserine, PI – phosphatidylinositol, PC – phosphatidylcholine, CER – ceramide, DG – diacylglycerol, TG – triacylglycerol, PA – phosphatidic acid, SM – sphingomyelin, PG – phosphatidylglycerol

ESI-MS/MS was used to determine the phospholipid profile of the cortex, hippocampus, and hypothalamus of the PLB4 hBACE1 knock-in murine model of AD. Analysis of phosphatidylglycerols (PG), phosphatidylethanolamines (PE, including *lyso*-phosphatidylethanolamines (LPE)) and sphingomyelins (SM) are shown in Fig. 3, phosphatidylcholines (PC, including *lyso*-phosphatidylcholines (LPC)), diacylglycerols (DG) and triacylglycerols (TG) in Fig. 4, phosphatidylinositols (PI, including *lyso*-phosphatidylinositols LPI)), phosphatidylserines (PS), phosphatidic acids (PA), ceramides (Cer) and other sphingolipids in Fig. 5, and the collective phospholipid profiles in Fig. 6. Results showed statistically significant alterations in PE, PC, PI, TG, DG, PS, Cer, SM, PG, and PA, listed in order of abundance of changes, the majority of which were observed in the PLB4 hippocampus, followed by the hypothalamus, and a few alterations were found in the cortex. A summary of the significant alterations between individual phospholipid species in the PLB_WT_ and PLB4 cortex, hippocampus, and hypothalamus samples, are listed in Supplementary Table 1.

Statistically significant alterations were observed in 30 different PE species including plasmalogen PE in the PLB4 hippocampus, and the PLB4 cortex, but only 5 PE species (4 of which were plasmalogen) in the PLB4 hypothalamus – compared to PLB_WT_ control (Fig. 3). *The observed changes within the plasmalogen PEs were the largest in the hippocampus, the changes are very specific depending upon acyl chain length, which may imply specific lipids may have specific roles within different sub-regions of the hippocampus.* The overall PE content showed a statistically significant decrease by 1.5-fold (*P* = 0.0189) in the PLB4 cortex compared to PLB_WT_, a decrease in PE content was also observed in the PLB4 hippocampus, and an increase in the PLB4 hypothalamus, but no statistical significance was observed (Fig. 6). While some individual PC species were increased in the PLB4 cortex (1 alteration) and hippocampus (12 alterations, predominantly increased), the PLB4 hypothalamus showed the biggest changes, with a total of 22 altered PC species – 2 increases and 20 decreases (Fig.4). The total PC content of the PLB4 hippocampus showed a statistically significant increase by 1.5-fold (*P* = 0.0469) compared to the PLB_WT_ hippocampus (Fig. 6).

Several alterations in PI species were detected across all three brain regions (Fig. 5): the PLB4 cortex showed the biggest changes, with altered levels of 9 PI species, followed by the PLB4 hypothalamus with 5 alterations, and the PLB4 hippocampus with 4 alterations. Whilst LPI (18:0) showed a consistent decrease across all three PLB4 brain regions, PI (36:4) was decreased in both the PLB4 cortex and hippocampus, but increased in the PLB4 hypothalamus – compared to PLB_WT_. Total PI levels were slightly decreased in both the PLB4 cortex and hippocampus, but showed a statistically significant increase in the PLB4 hypothalamus by 1.5-fold (*P* = 0.0444) compared to PLB_WT_ (Fig. 6). Many TG species were decreased in the PLB4 hypothalamus (6 changes), PLB4 cortex (4 changes) and PLB4 hippocampus (3 changes; Fig. 4). The most pronounced alterations were observed in TG (58:12), TG (62:14), and TG (66:18) – which were all depleted in the PLB4 hypothalamus compared to the PLB_WT_ hypothalamus. The total TG content showed a statistically significant decrease in the PLB4 hippocampus, by 1.5-fold (*P* = 0.0009), as well as in the PLB4 hypothalamus by 3.1-fold (*P* = 0.0090) – compared to PLB_WT_ (Fig. 6). Across the panel of 7 DG species identified (Fig. 4), many showed an increase in both the PLB4 cortex and hippocampus, with statistically significant increases observed in 3 DG species in the PLB4 cortex and 1 DG species in the PLB4 hippocampus. However, the PLB4 hypothalamus displayed the opposite trend, showing decreased levels of several DG species. In particular, the most prominent change was observed in DG (42:10), which was completely depleted in the PLB4 hypothalamus compared to the PLB_WT_ hypothalamus, but increased in the PLB4 cortex and hippocampus. Overall, the total DG levels showed a statistically significant increase in the PLB4 hippocampus by 1.4-fold (*P* = 0.0378) compared to PLB_WT_ (Fig. 6).

The majority of the PS alterations were observed in the PLB4 hypothalamus (Fig. 5), where 4 PS species showed statistically significant decreases compared to PLB_WT_, with one increase observed in the PLB4 hippocampus, and no statistically significant changes found in the cortex. Whilst total PS levels showed a decrease across all three PLB4 brain regions, no statistically significant alterations were observed (Fig. 6). Some statistically significant alterations were observed in the Cer species in the PLB4 hippocampus (2 changes) and hypothalamus (3 changes), but none in the cortex (Fig. 5). No statistically significant alterations were detected in the total Cer content of either the PLB4 cortex, hippocampus, or cerebellum (Fig. 6). Of the 7 SM species identified (Fig. 3), only 1 was decreased in the PLB4 cortex, while 1 was increased in the PLB4 hypothalamus, compared to PLB_WT_. The total SM content showed a slight decrease in both the PLB4 cortex and hippocampus, and a slight increase in the PLB4 hypothalamus, but no statistically significant differences were detected (Fig. 6). A modest increase in the major monogalactosylceramides, identified as HexCer (d36:2), in both the hypothalamus and hippocampus (Fig. 5) were observed. Three different gangliosides were identified (Fig. 5), but their levels did not seem significantly altered in any of the brain regions of the mouse PLB4 model, compared to wild-type.

Whilst several PG species were detected across the PLB_WT_ and PLB4 samples (Fig. 3), only PG (38:0) showed a statistically significant increase in the PLB4 cortex compared to the PLB_WT_ cortex, and no statistically significant changes were detected in the total PG content in any of the three brain regions (Fig. 6). No statistically significant alterations were observed in the PA levels between the PLB_WT_ and PLB4 brain regions (Figs. 5 and 6).

## Discussion

Lipidomics is a powerful analytical platform, which allows extensive analysis of the multifaceted lipidome. The combinatory use of GC-MS and ESI-MS/MS enabled the detection, identification, and analysis of a range of sterols, fatty acids, and phospholipids in brain regions relevant to the pathogenesis of AD, highlighting alterations in specific lipids. This comprehensive quantitative lipidomics analysis identified a total of forty-nine lipid alterations in the cortex, sixty in the hippocampus, and fifty-one in the hypothalamus of the PLB4 mouse brain, as summarised in Table 1, thus providing detailed insight into the consequences of the targeted neuronal knock-in of hBACE1 upon phospholipid, sterol, and fatty acid levels within the three brain regions. In addition to detecting overall changes in lipid classes, the lipidomics approach identified specific lipid species that are altered within the corresponding groups, allowing for a thorough, quantitative comparison between the PLB_WT_ and PLB4 mouse brain regions. The results suggest disruptions in key lipid metabolism pathways – furthermore, these disruptions may be highly localised, thus supporting the notion of selective vulnerability of certain brain regions to AD pathogenesis during the progression of the disease.

**Table 1:**
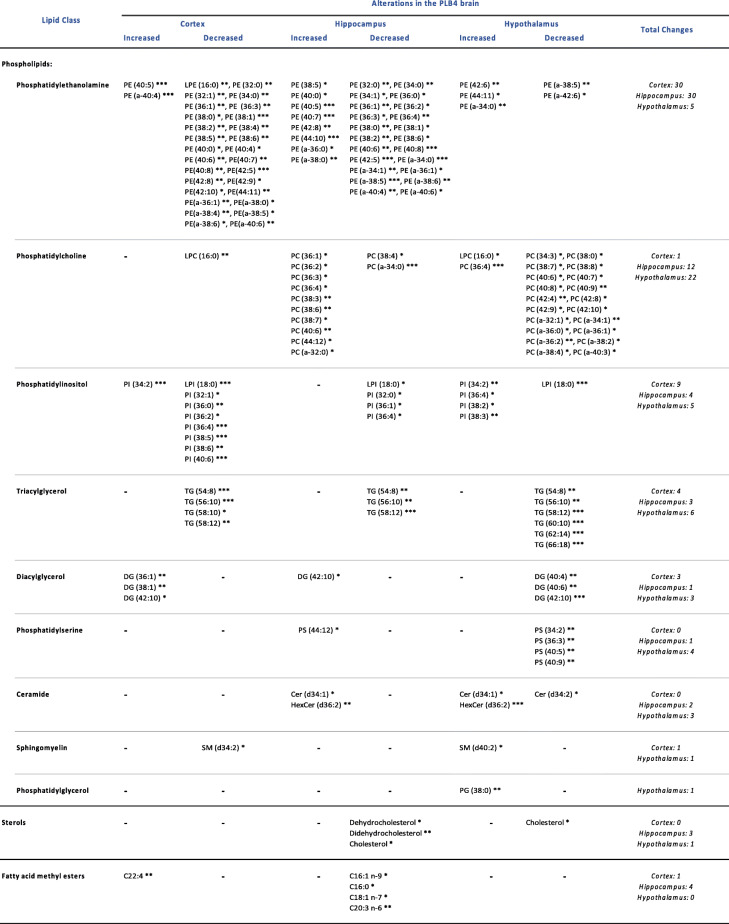
Summary of the lipid alterations in the PLB4 cortex, hippocampus, and hypothalamus. Lipid species listed by class showing the most the statistically significant alterations in the PLB4 brain compared to PLB_WT_. An unpaired, two-tailed Student’s T-test with Welch’s correction was performed to determine statistically significant differences between PLBWT and PLB4 lipid species and brain regions: * *p* ≤ 0.05, ** *p* ≤ 0.01, *** *p* ≤ 0.001. Abbreviations: PE – phosphatidylethanolamine, PC – phosphatidylcholine, PI – phosphatidylinositol, TG – triacylglycerol, DG – diacylglycerol, PS – phosphatidylserine, Cer – ceramide, SM – sphingomyelin, PG – phosphatidylglycerol

The PLB4 model displays several disease relevant phenotypes, and recapitulates the early stages of AD due to the increased production and aggregation of Aβ_40/42_, without the formation of end-stage Aβ plaques [32]. Therefore, the model offers an opportunity to observe early alterations within the murine brain that arise due to low level expression of the hBACE1 enzyme and the resultant increase in Aβ production. In the human AD brain, tau pathology, rather than Aβ pathology, is strongly associated with disease progression, and neuropathological studies show that the burden of tau neurofibrillary tangles, but not Aβ pathology, correlates with the degree of cognitive impairment [33, 34]. Notably however, the PLB4 hBACE1 AD mouse model displays memory impairments at 6 months of age, despite the lack of overt tau pathology within the brain [21], therefore suggesting that the hBACE1 knock-in and metabolic dysfunction may have a role in influencing the cognitive deficits observed. Impairments in cerebral metabolism have been linked to cognitive decline in AD [35, 36], thus, the role of BACE1 in metabolic regulation and the diabetic phenotype observed in the PLB4 hBACE1 knock-in mouse model may contribute to memory impairment. Cholesterol-based microdomains of the phospholipid bilayer at synapses influence several essential aspects of neurotransmission, ranging from regulation of synaptic protein activity, to vesicle fusion, as well as synapse formation and stability [37, 38]. Consequently, a potential cause for the cognitive symptoms seen in the PLB4 hBACE1 knock-in mice may be the alterations in cholesterol regulation within the brain, as highlighted by the results from the lipidomics analysis. Additionally, analysis of the phospholipids within the PLB4 hippocampus revealed an increase in the total PC content, which is a precursor and suggested reservoir for choline within the cholinergic system [39]. Cholinergic deficits in the AD brain are strongly linked to cognitive impairment, and thus, the alterations in the PC content of the PLB4 hippocampus may suggest underlying modifications in cholinergic neurotransmission within the brain.

Analysis of the fatty acid profile of the PLB4 hBACE1 knock-in mouse brain highlighted many alterations within the hippocampus, thus suggesting a potential role for BACE1 in the pathological mechanisms influencing fatty acid metabolism in AD. Analysis of the phospholipid profiles in the PLB4 brain indicate alterations in many PE species, which is the second most abundant phospholipid in the mammalian cell [40], and is directly associated with mitochondrial membrane dynamics, stability and function [40]. PE metabolism is linked with PS metabolism via the PS decarboxylase pathway [40], and thus may indicate an interconnected mechanism for the altered PS levels observed in the PLB4 hippocampus. The metabolism of PI, which showed many alterations in the PLB4 brain, is linked with a multitude of signalling pathways, including protein kinase C (PKC) activation, and calcium signalling, with implications upon cell motility, adhesion, and survival [41], as well several key aspects of neurotransmission [42], hence the decreased levels of PI within the PLB4 brain could have widespread downstream effects.

An important influence of lipid dysregulation observed within the various brain regions in the AD-like mouse model is upon neurotransmission via lipid-protein interactions at synapses. Lipids, both within the cell membrane and those involved in signalling cascades, play an essential role to regulate the activity of synaptic proteins, as well as the coordination of exo- and endocytotic fusion of synaptic vesicles. In AD, synaptic dysfunction has been associated with altered post-synaptic density scaffolds and glutamatergic neurotransmission, which in turn have been linked to alterations in the lipid composition of subsynaptic protein domains via membrane lipid rafts [28–30].

The significant reduction in the neutral lipids in the hypothalamus does suggest that they may be utilised as an energy source, i.e. fatty acid beta-oxidation, which would fit well with the hypothesis that a reduction in glycolysis is associated with ageing of the brain and, in extreme cases, the catabolism of structural proteins could result in irreversible damage, leading to several types of dementia including AD, as they are involved in several complex signalling networks, including PKC activation and calcium-mediated vesicle fusion [43], as well as synaptic modulation via glutamate receptors [44]. Furthermore, a recent study by Wood et al. reported elevated DG levels in both the frontal cortex and plasma samples of subjects with MCI, and proposed that the accumulation of DG, as observed here in the cortex and hippocampus, occurs early during the pathophysiological progression of AD [45]. Additionally, increased cellular DG content has also been linked to insulin resistance via the activation of PKC, which consequently influences glycogen synthesis [32].

Several phospholipid groups in the forebrain and plasma of PLB4 hBACE1 knock-in mice were previously assessed by the Folch lipid extraction technique and LC/MS [25] – interestingly, many lipid classes were predominantly increased in the PLB4 forebrain. Comparatively, the results from this study, which used the Bligh and Dyer lipid extraction methodology [31] and ESI/MS-MS to analyse brain region-specific phospholipid alterations showed some similarities. However, these results predominantly showed decreased levels of several minor, but important, phospholipid species across the cortex, hippocampus, and hypothalamus of the PLB4 brain compared to PLB_WT_. Possible reasons for the variations observed could be due to the analysis of the lipid content of the entire PLB4 forebrain in the study by Plucińska et al. [25] compared to that of specific PLB4 brain regions in the current study, as well as differences in the lipid extraction technique and quantitative lipidomics analyses. A similar comparative lipidomics study was performed by Chan et al. [46], which investigated the lipid profile of the forebrain in three commonly used familial AD murine models: the PSEN1_M146V_ (PS1) model [47], the APP_K670N,M671L_ Swedish mutant (APP_Swe_) [48], and the PS1-APP double transgenic model [49], between the ages of 9–11.5 months, using the Bligh and Dyer lipid extraction method, and LC/MS. As with our analysis, the PS1 single transgenic AD mouse model showed significant decreases in the PS and PI content of the forebrain, as did the APP_Swe_ mouse model, which additionally displayed an overall increase in PC levels. Many of these changes were also seen in the PS1-APP mouse forebrain, and the authors proposed a potential synergistic effect of the two transgenes. The study by Chan et al. also compared the lipid profiles of the prefrontal cortex, entorhinal cortex, and cerebellum from human post mortem late-onset AD cases, noting that, on average, the entorhinal cortex exhibited higher levels of neuropathology than the prefrontal cortex. Results from their analysis showed that the prefrontal cortex and entorhinal cortex lipid profiles showed abundant alterations, such as decreased levels of PE, and increased levels of Cer, DG, and SM, amongst others sphingolipids, which was also observe in this study.

While there are numerous caveats to comparisons between various transgenic AD mouse models, including even the different WT background from which the transgenic murine lines are derived, and further to post mortem human AD brain tissue, the results from the hBACE1 knock-in mouse model of AD supports the concept of brain region – specific alterations in lipid metabolism. This comprehensive lipidomics analysis indicates dysregulation of various lipid metabolism pathways, with brain region specificity, and furthermore could suggest the pathological dysregulation of key enzymes integral to the regulation of lipid homeostasis within the brain. Whilst further investigations need to be undertaken to assess whether the lipid alterations we observed are cause or consequence of the ‘AD-like’ and diabetic phenotype of the PLB4 hBACE1 knock-in mouse model, the findings highlight potential lipid groups that warrant further investigations. The importance of delineating the mechanisms contributing to aberrant lipid homeostasis in AD is highlighted by research suggesting the use of lipid biomarkers to distinguish between MCI and AD [18–20].

The results here, alongside several other lipidomics studies, show the importance of assessing brain-region specific lipid alterations, and lays the foundation for further investigations into changes within specific lipid metabolism pathways. In particular, it would be interesting to explore the alterations in lipid metabolism pathways at the protein level, and whether the inhibition of BACE1 reverted the lipid alterations observed towards a WT profile. Additionally, considering the region-specific variations seen in lipid classes and specific lipid species within the PLB4 brain, investigating the metabolic interdependence of different cell types within the brain regions could also provide valuable insight.

Recent advances in mass spectrometry techniques, such as imaging have for the first time allowed direct probing of brain sections/regions. These have clearly highlighted specific alterations of a range of lipids in AD brains and plaques [50–52]. However, advances in improving the absolute resolution of the brain area that can be queried will allow small sub-regions of the brains to be analyzed, One of the drawbacks of these direct methods is ion suppression by those species that ionize well, i.e. phosphatidylcholine, over neutral lipids. Coupled with a lack of the ability of spiking internal standards, means that absolute quantification is as yet not possible.

### Study strengths and limitations

The strengths of this study are that the results show extensive brain region-specific phospholipid alterations, as well as several sterol and fatty acid changes in the PLB4 brain, which indicate modifications in lipid homeostasis and likely several lipid metabolic pathways relevant to the pathogenesis of AD. This PLB4 mouse model, in conjunction with potential therapies could allow a readout both from behaviours, but also from readdressing the lipid “miss-use”, i.e. catabolism of lipids by an “AD-like” brain. However, the limitations of any type of lipidomic or metabolomic study of this nature, is the fact that it is only a model, and the data obtained is only a snap shot for this type of analysis, telling us nothing about the actual rate/flux of certain metabolic functions/pathways.

## Conclusion

In conclusion, several significant brain-region specific alterations were found within many major and minor lipid groups in the PLB4 hBACE1 knock-in mouse brain, many of which were observed predominantly in the hippocampus and hypothalamus, which is in concordance to the neuropathological and clinical progression of AD, and highlights the relevance of this mouse model for AD research. Collectively, these results are consistent with the hypothesis of regional vulnerability of the brain to lipid dysregulation, and supports the premise that metabolic abnormalities have an important role in both AD and diabetes, where a carbon source usage is important for fuelling the brain. While this study has identified important alterations within key lipid species, further research is needed to gain more detailed understanding of the biochemical and mechanistic alterations associated with the lipid dysregulation observed in AD. A future perspective of this work is the use of this mouse model as a way to evaluate possible therapies to readdress the lipid dysregulation and imbalance observed.

## Supplementary information

**Additional file 1: Supplementary Table S1.** Summary of the lipid alterations in the PLB4 cortex, hippocampus, and hypothalamus. Lipid species listed show all the statistically significant alterations in the PLB4 brain compared to PLBWT. An unpaired, two-tailed Student’s T-test with Welch’s correction was performed using the average cpm data to determine statistically significant differences between PLBWT and PLB4 lipid species and brain regions: * *p* ≤ 0.05, ** *p* ≤ 0.01, *** *p* ≤ 0.001. A positive (+) percentage change denotes an increase in PLB4 compared to PLB_WT_, a negative (−) percentage change denotes a decrease in PLB4 compared to PLBWT. Abbreviations: cpm: counts per million, PE – phosphatidylethanolamine, PC – phosphatidylcholine, PI – phosphatidylinositol, TG – triacylglycerol, DG – diacylglycerol, PS – phosphatidylserine, Cer – ceramide, SM – sphingomyelin, PG – phosphatidylglycerol.

**Additional file 2.**

**Additional file 3.**

**Additional file 4.**

**Additional file 5.**

## Data Availability

The datasets generated and analysed during the current study are available from the corresponding author upon reasonable request. As well as being made publically available on a suitable database.

## Abbreviations

AD: Alzheimer’s disease
Aβ: amyloid-β
ApoE: apolipoprotein E
APP: amyloid precursor protein
BACE1: β-site cleavage enzyme 1
ESI-MS/MS: electrospray ionisation - tandem mass spectrometry
GC-MS: gas chromatography - mass spectrometry
PG: phosphatidylglycerol
LPE: *lyso*-phosphatidylethanolamine
PE: phosphatidylethanolamine
SM: Sphingomyelin
LPC: *lyso*-phosphatidylcholine
PC: phosphatidylcholine
DG: diacylglycerol
TG: triacylglycerol
LPI: *lyso*-phosphatidylinositol
PI: phosphatidylinositol
PA: phosphatidic acid
Cer: ceramide
MCI: mild cognitive impairment
BBB: blood brain barrier
CNS: central nervous system
