# Supplementary figures and images for: Brain region–specific lipid alterations in the PLB4 hBACE1 knock-in mouse model of Alzheimer’s disease
Source: Lipids Health Dis. 2020 Aug 31;19:201. doi: 10.1186/s12944-020-01367-8 (PMC7457777; doi:10.1186/s12944-020-01367-8)

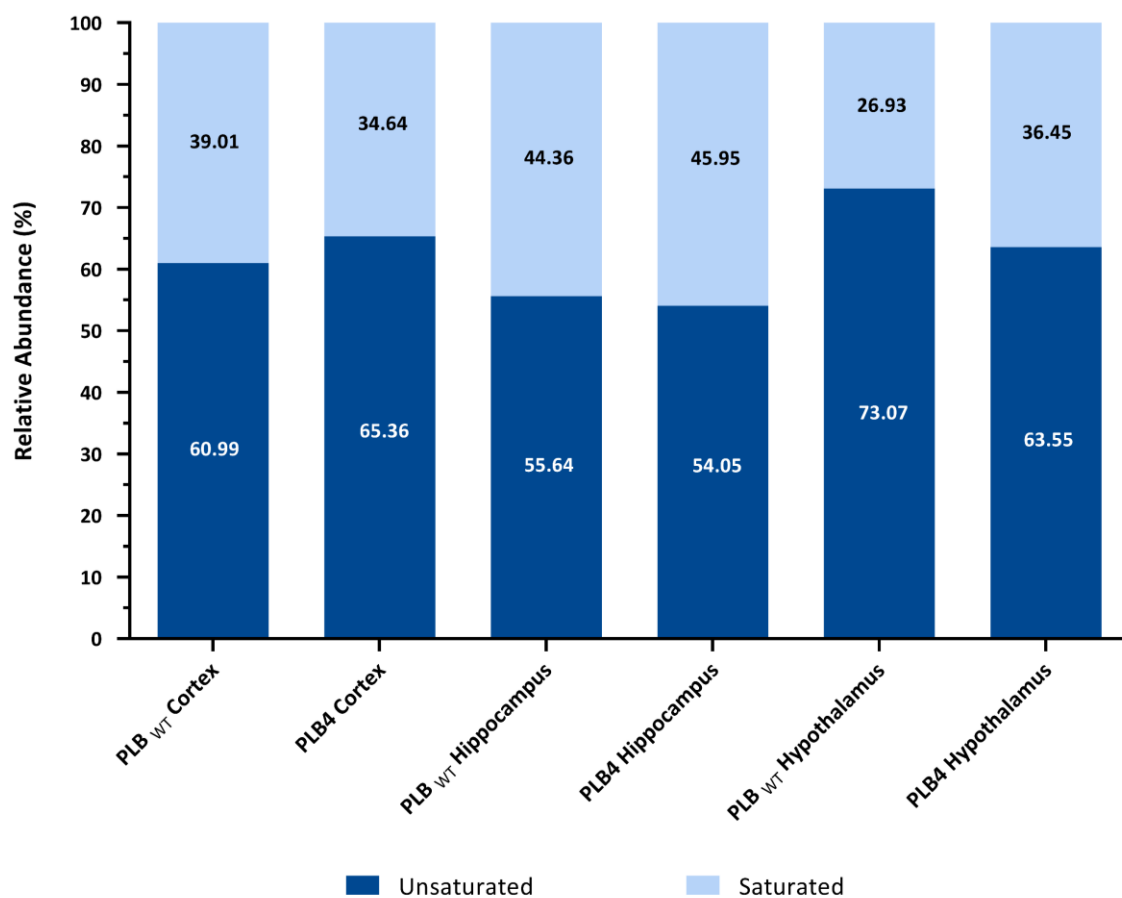

Supplement: Supplementary file 2 — Additional file 2. [file 12944_2020_1367_MOESM2_ESM.pdf]

S3a.

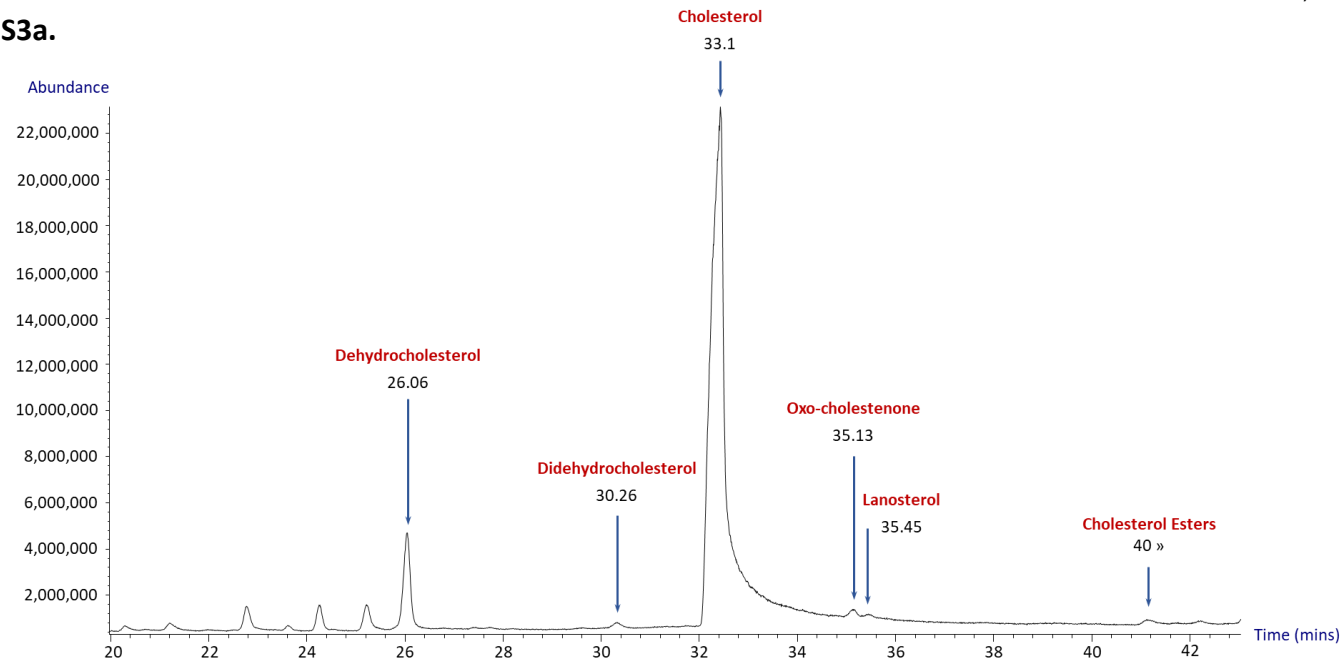

S3b.

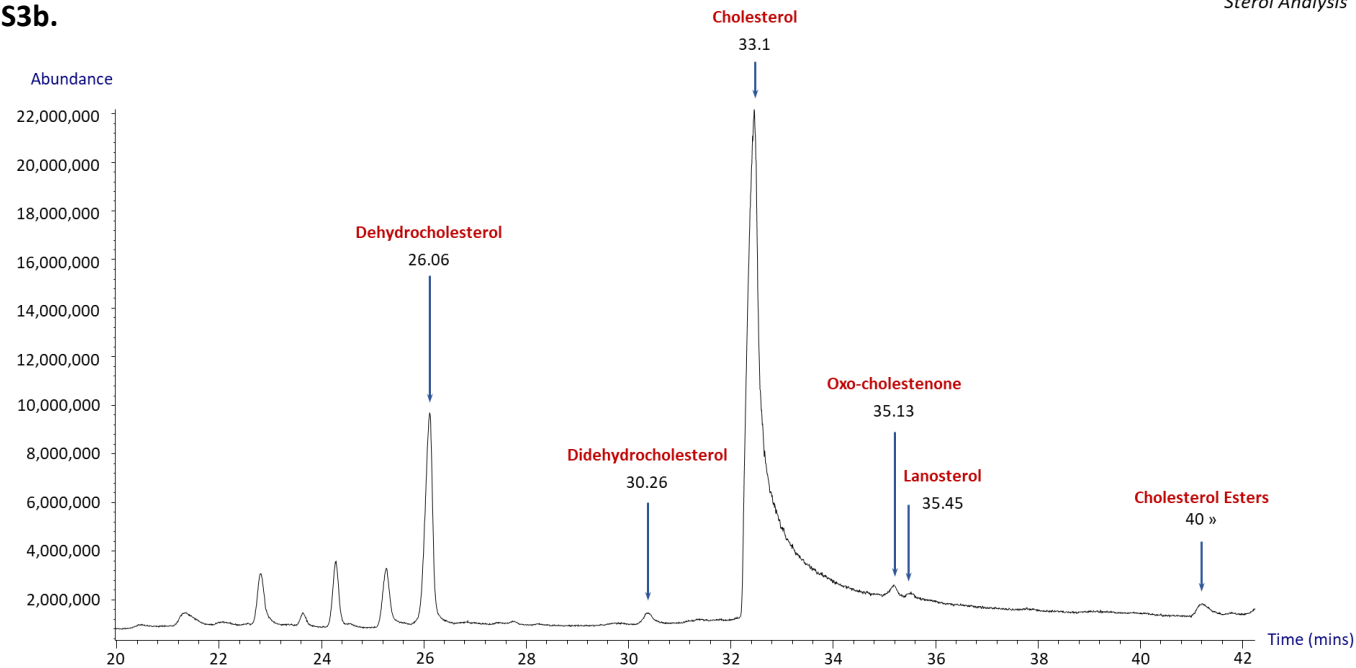

S3c.

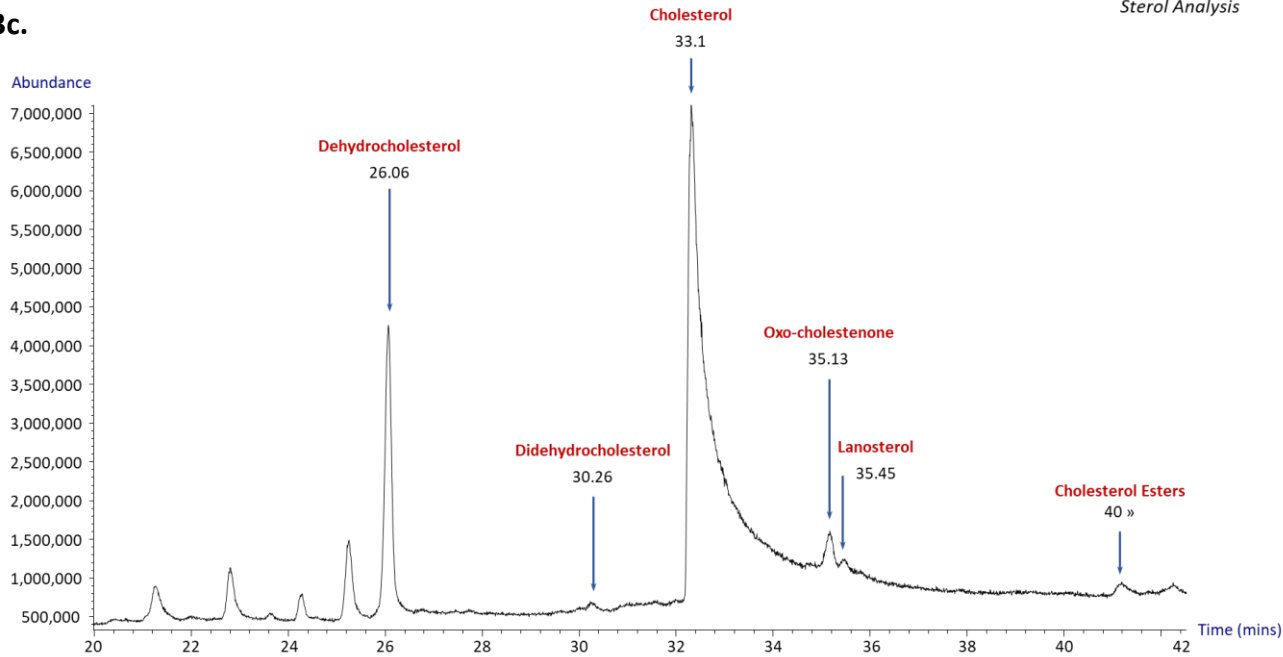

S3d.

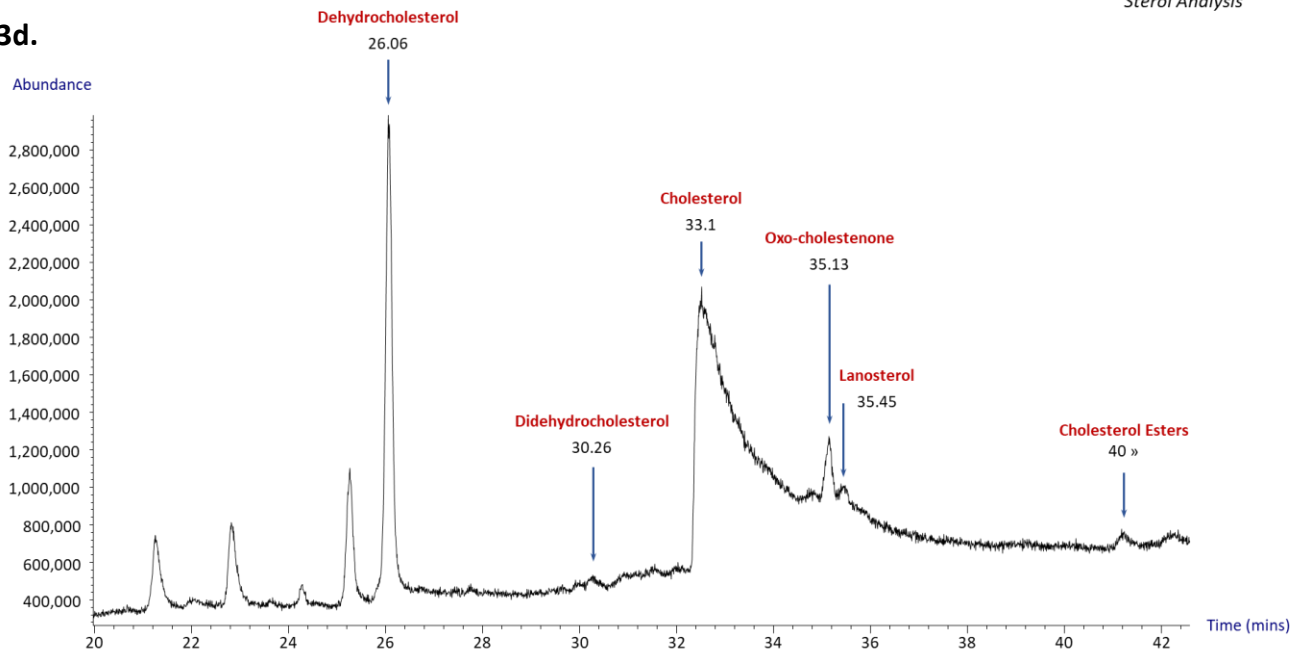

S3e.

WT HYPOTHALAMUS  
Sterol Analysis

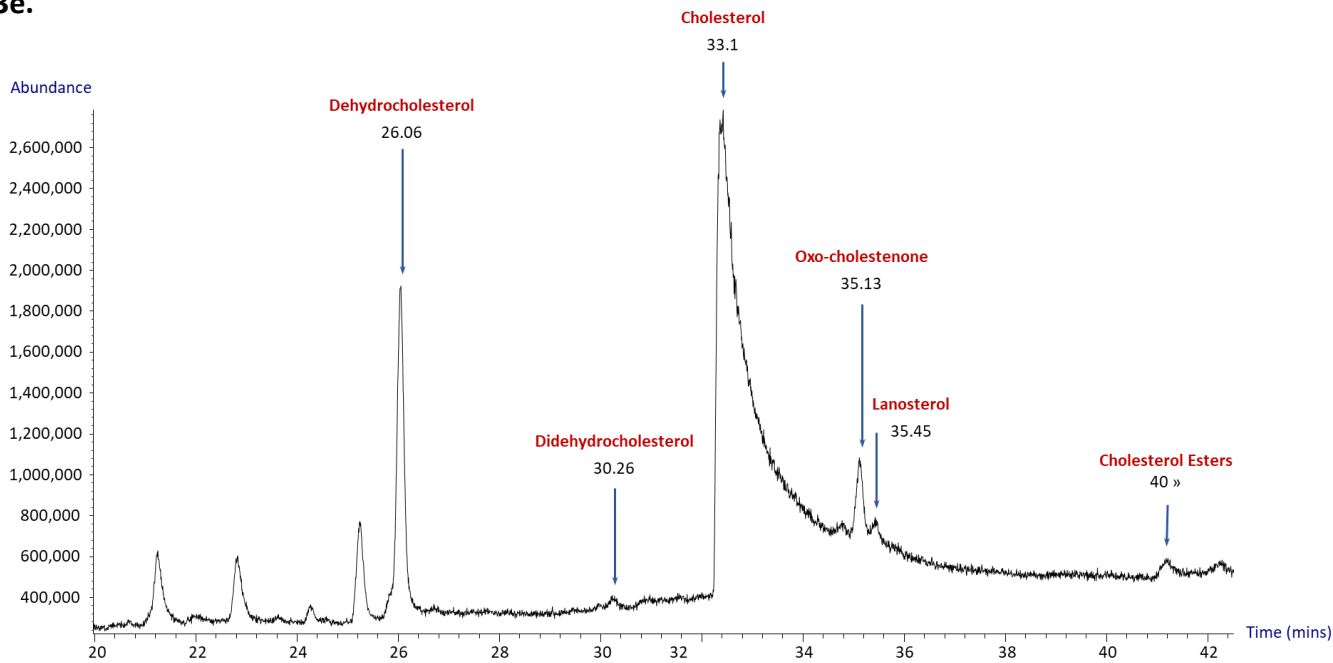

S3f.

PLB4 HYPOTHALAMUS  
Sterol Analysis

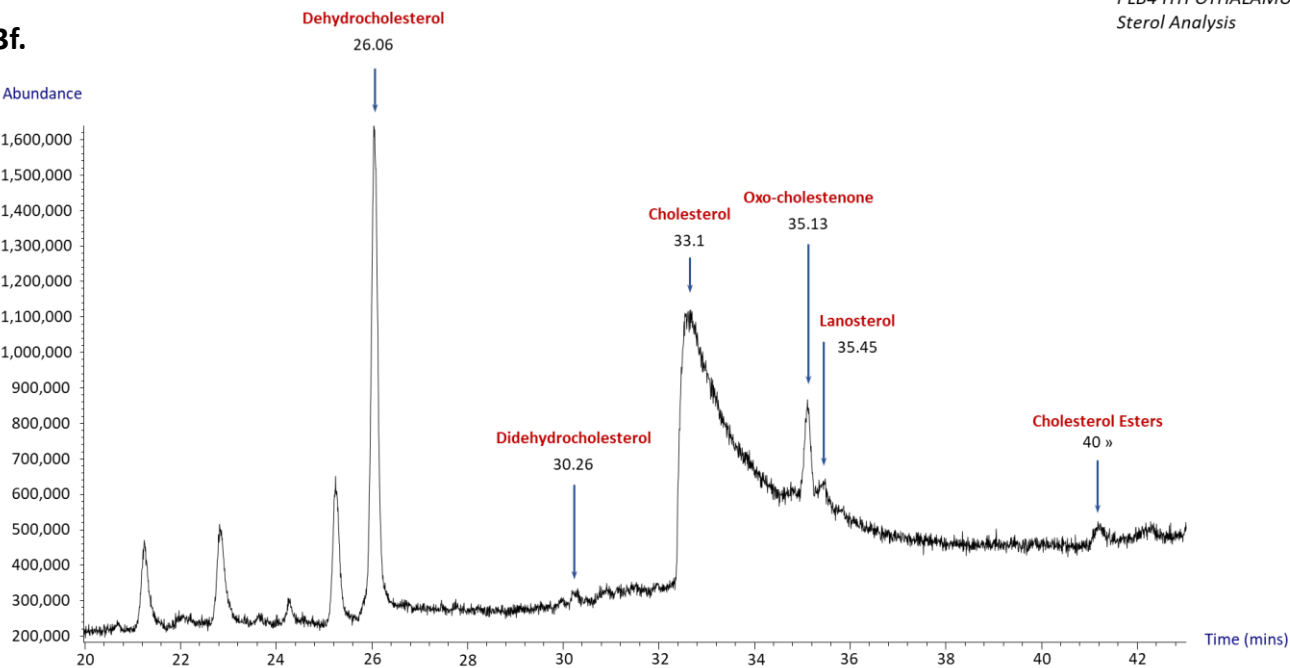

Supplement: Supplementary file 4 — Additional file 4. [file 12944_2020_1367_MOESM4_ESM.pdf]
