# Supplementary material for: Brain region–specific lipid alterations in the PLB4 hBACE1 knock-in mouse model of Alzheimer’s disease
Source: Lipids Health Dis. 2020 Aug 31;19:201. doi: 10.1186/s12944-020-01367-8 (PMC7457777; doi:10.1186/s12944-020-01367-8)

S2a.

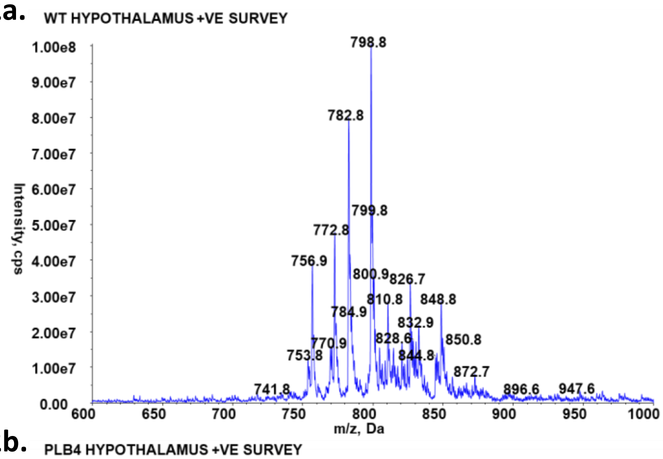

S2b.

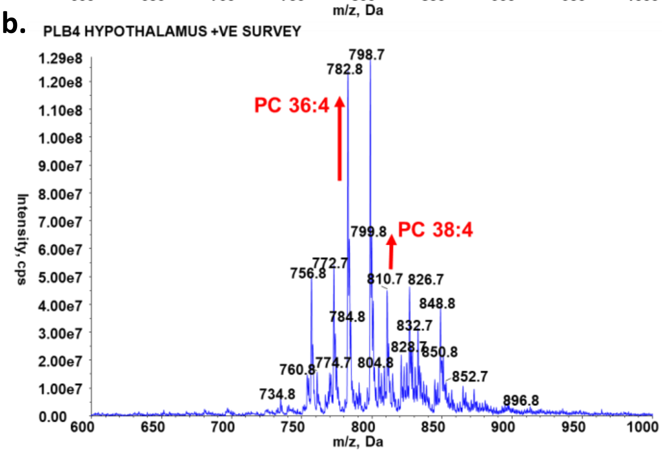

S2c.

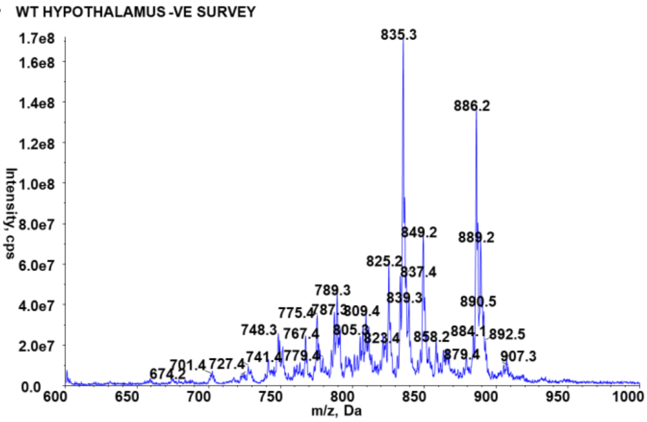

S2d.

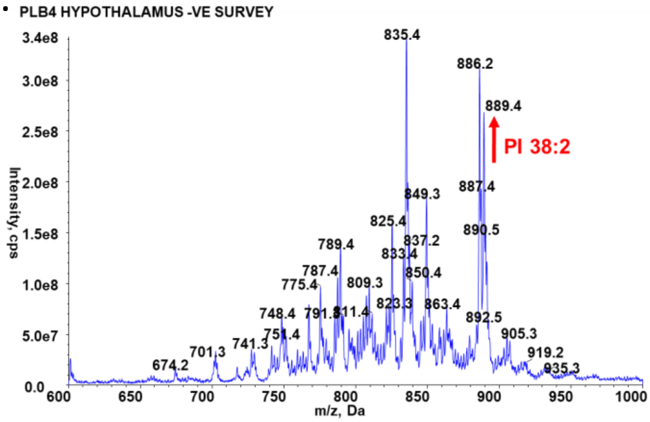

Supplement: Supplementary file 3 — Additional file 3. [file 12944_2020_1367_MOESM3_ESM.pdf]
