# Supplementary material for: Brain region–specific lipid alterations in the PLB4 hBACE1 knock-in mouse model of Alzheimer’s disease
Source: Lipids Health Dis. 2020 Aug 31;19:201. doi: 10.1186/s12944-020-01367-8 (PMC7457777; doi:10.1186/s12944-020-01367-8)
Supplement: Supplementary file 5 — Additional file 5. [file 12944_2020_1367_MOESM5_ESM.pdf]

S4a.

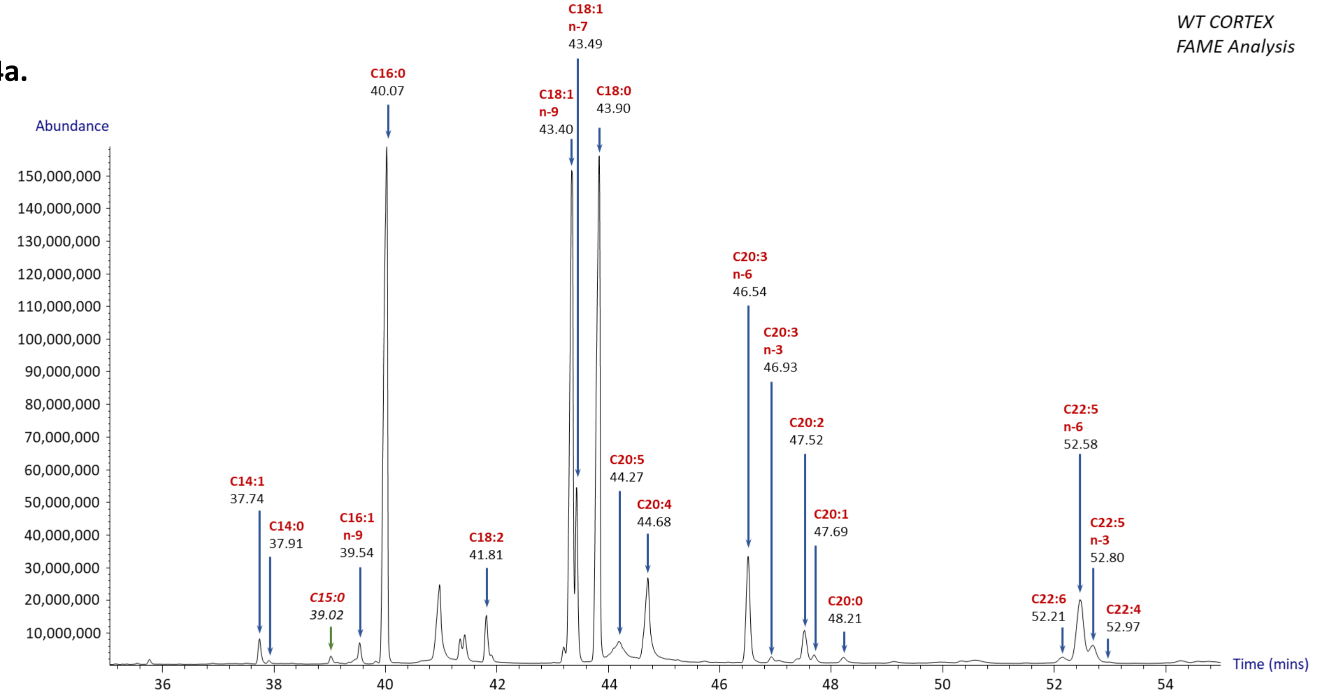
WT CORTEX
FAME Analysis
Abundance
Time (mins)
C14:1 37.74
C14:0 37.91
C15:0 39.02
C16:1 n-9 39.54
C16:0 40.07
C18:2 41.81
C18:1 n-9 43.40
C18:1 n-7 43.49
C18:0 43.90
C20:5 44.27
C20:4 44.68
C20:3 n-6 46.54
C20:3 n-3 46.93
C20:2 47.52
C20:1 47.69
C20:0 48.21
C22:6 52.21
C22:5 n-6 52.58
C22:5 n-3 52.80
C22:4 52.97

S4b.

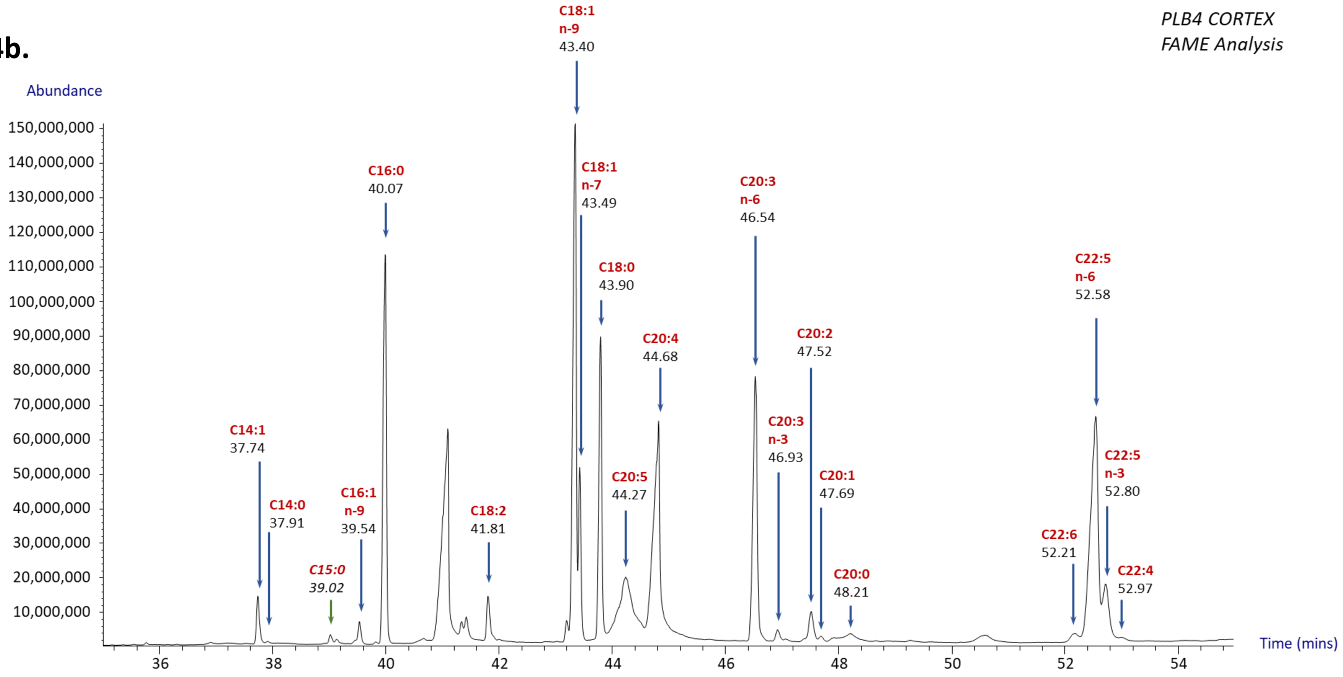
PLB4 CORTEX
FAME Analysis
Abundance
Time (mins)
C14:1 37.74
C14:0 37.91
C15:0 39.02
C16:1 n-9 39.54
C16:0 40.07
C18:2 41.81
C18:1 n-9 43.40
C18:1 n-7 43.49
C18:0 43.90
C20:5 44.27
C20:4 44.68
C20:3 n-6 46.54
C20:3 n-3 46.93
C20:2 47.52
C20:1 47.69
C20:0 48.21
C22:6 52.21
C22:5 n-6 52.58
C22:5 n-3 52.80
C22:4 52.97

S4c.

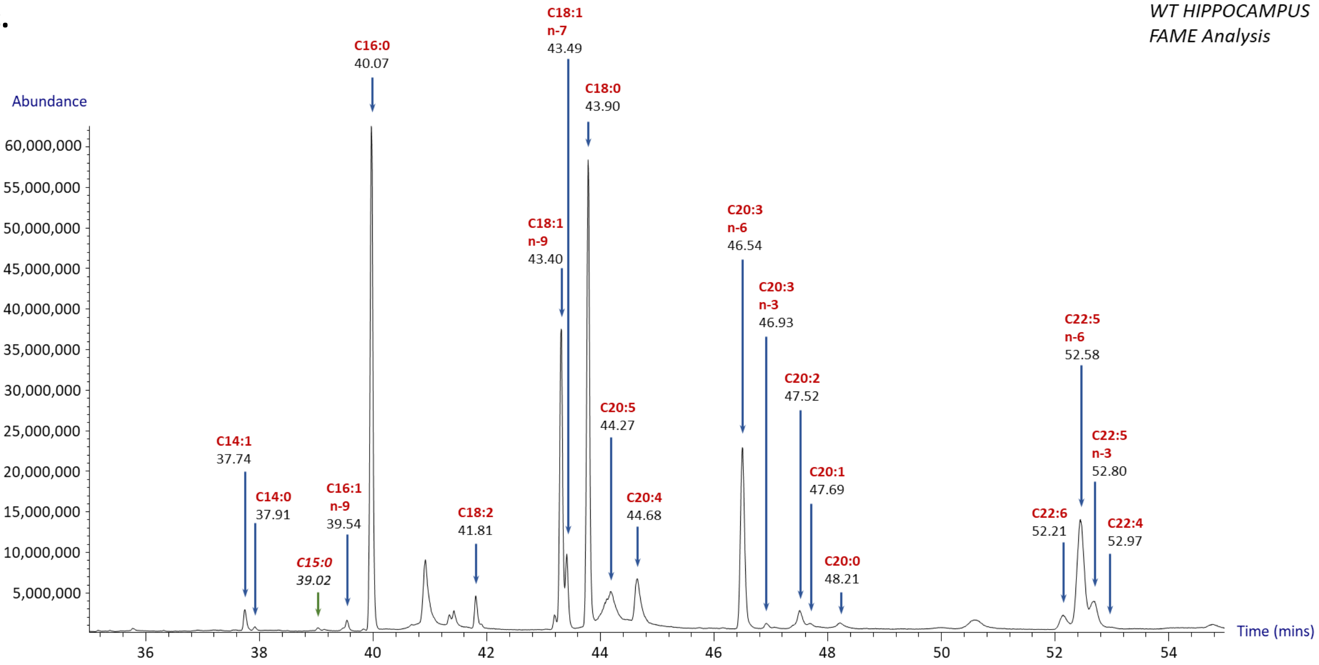
WT HIPPOCAMPUS
FAME Analysis
Abundance
60,000,000
55,000,000
50,000,000
45,000,000
40,000,000
35,000,000
30,000,000
25,000,000
20,000,000
15,000,000
10,000,000
5,000,000
36
38
40
42
44
46
48
50
52
54
Time (mins)
C14:1 37.74
C14:0 37.91
C15:0 39.02
C16:1 n-9 39.54
C16:0 40.07
C18:2 41.81
C18:1 n-9 43.40
C18:1 n-7 43.49
C18:0 43.90
C20:5 44.27
C20:4 44.68
C20:3 n-6 46.54
C20:3 n-3 46.93
C20:2 47.52
C20:1 47.69
C20:0 48.21
C22:6 52.21
C22:5 n-6 52.58
C22:5 n-3 52.80
C22:4 52.97

S4d.

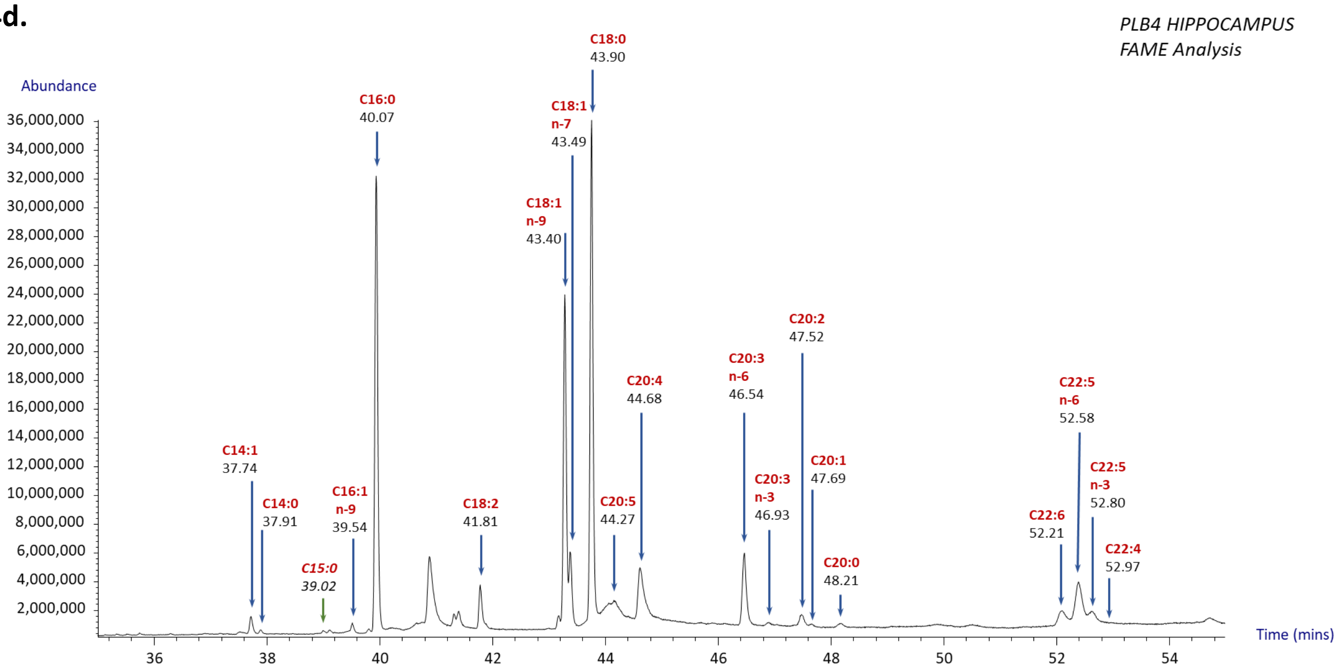
PLB4 HIPPOCAMPUS
FAME Analysis
Abundance
36,000,000
34,000,000
32,000,000
30,000,000
28,000,000
26,000,000
24,000,000
22,000,000
20,000,000
18,000,000
16,000,000
14,000,000
12,000,000
10,000,000
8,000,000
6,000,000
4,000,000
2,000,000
36
38
40
42
44
46
48
50
52
54
Time (mins)
C14:1 37.74
C14:0 37.91
C15:0 39.02
C16:1 n-9 39.54
C16:0 40.07
C18:2 41.81
C18:1 n-9 43.40
C18:1 n-7 43.49
C18:0 43.90
C20:5 44.27
C20:4 44.68
C20:3 n-6 46.54
C20:3 n-3 46.93
C20:2 47.52
C20:1 47.69
C20:0 48.21
C22:6 52.21
C22:5 n-6 52.58
C22:5 n-3 52.80
C22:4 52.97

S4e.

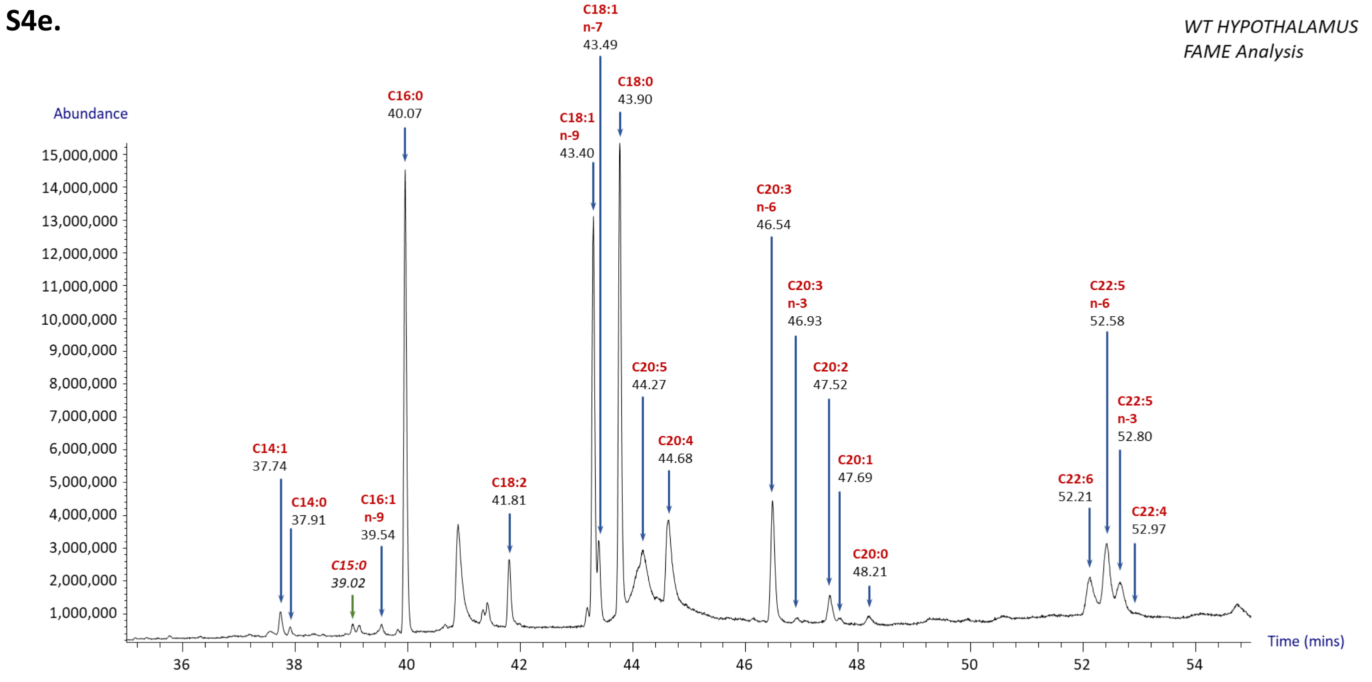
WT HYPOTHALAMUS
FAME Analysis
Abundance
15,000,000
14,000,000
13,000,000
12,000,000
11,000,000
10,000,000
9,000,000
8,000,000
7,000,000
6,000,000
5,000,000
4,000,000
3,000,000
2,000,000
1,000,000
C14:1 37.74
C14:0 37.91
C15:0 39.02
C16:1 n-9 39.54
C16:0 40.07
C18:2 41.81
C18:1 n-9 43.40
C18:1 n-7 43.49
C18:0 43.90
C20:5 44.27
C20:4 44.68
C20:3 n-6 46.54
C20:3 n-3 46.93
C20:2 47.52
C20:1 47.69
C20:0 48.21
C22:6 52.21
C22:5 n-6 52.58
C22:5 n-3 52.80
C22:4 52.97
36
38
40
42
44
46
48
50
52
54
Time (mins)

S4f.

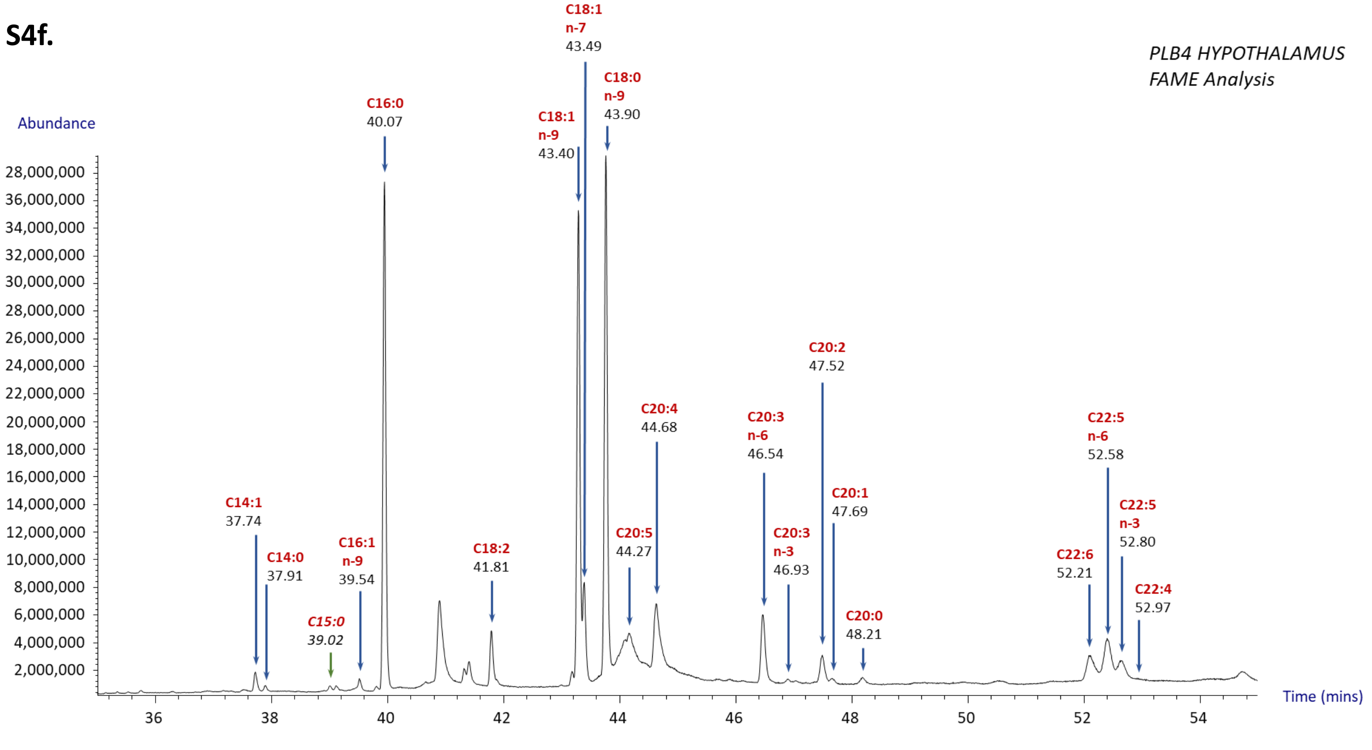
PLB4 HYPOTHALAMUS
FAME Analysis
Abundance
28,000,000
36,000,000
34,000,000
32,000,000
30,000,000
28,000,000
26,000,000
24,000,000
22,000,000
20,000,000
18,000,000
16,000,000
14,000,000
12,000,000
10,000,000
8,000,000
6,000,000
4,000,000
2,000,000
C14:1 37.74
C14:0 37.91
C15:0 39.02
C16:1 n-9 39.54
C16:0 40.07
C18:2 41.81
C18:1 n-9 43.40
C18:1 n-7 43.49
C18:0 n-9 43.90
C20:5 44.27
C20:4 44.68
C20:3 n-6 46.54
C20:3 n-3 46.93
C20:2 47.52
C20:1 47.69
C20:0 48.21
C22:6 52.21
C22:5 n-6 52.58
C22:5 n-3 52.80
C22:4 52.97
36
38
40
42
44
46
48
50
52
54
Time (mins)
